# Kinetics of Nucleo- and Spike Protein-Specific Immunoglobulin G and of Virus-Neutralizing Antibodies after SARS-CoV-2 Infection

**DOI:** 10.3390/microorganisms8101572

**Published:** 2020-10-13

**Authors:** Annabelle Strömer, Ruben Rose, Olaf Grobe, Franziska Neumann, Helmut Fickenscher, Thomas Lorentz, Andi Krumbholz

**Affiliations:** 1 Institute for Infection Medicine, Christian-Albrecht University and University Medical Center Schleswig-Holstein, Brunswiker Str. 4, 24105 Kiel, Germany; annabelle.stroemer@gmail.com (A.S.); rose@infmed.uni-kiel.de (R.R.); fickenscher@infmed.uni-kiel.de (H.F.); 2 Labor Dr. Krause und Kollegen MVZ GmbH, Steenbeker Weg 23, 24106 Kiel, Germany; grobe@labor-krause.de (O.G.); neumann@labor-krause.de (F.N.); lorentz@labor-krause.de (T.L.)

**Keywords:** COVID-19, humoral immunity, test performance, neutralization, follow-up, titer decrease

## Abstract

Kinetics of neutralizing antibodies and immunoglobulin G (IgG) against the nucleo (N) or spike (S) proteins of *severe acute respiratory syndrome coronavirus type* *2* (SARS-CoV-2) were studied in patients up to 165 days after PCR diagnosis of infection. Two immunoassays were selected out of eight IgG or total antibody tests by comparing their specificities and sensitivities. Sensitivities were calculated with convalescent sera from 26 PCR-confirmed cases, of which 76.9% had neutralizing antibodies (>1:10). Stored sera collected during the summer 2018 (N = 50) and winter seasons 2018/2019 (N = 50) were included to demonstrate the test specificities. IgG kinetics, avidities, and virus-neutralizing capacities were recorded over up to 165 days in eleven patients and five individuals from routine diagnostics. Sensitivities, specificities, and diagnostic accuracies ranged between 80.8–96.3%, 96.0–100%, and 93.7–99.2%, respectively. Nearly all results were confirmed with two different SARS-CoV-2-specific immunoblots. Six (54.4%) patients exhibited stable N-specific IgG indices over 120 days and longer; three of them developed IgG of high avidity. The S-specific IgG response was stable in ten (91.0%) patients, and eight (72.7%) had neutralizing antibodies. However, the titers were relatively low, suggesting that sustained humoral immunity is uncertain, especially after outpatient SARS-CoV-2 infection.

## 1. Introduction

By the end of the year, 2019, Chinese local health authorities reported the occurrence of a cluster of pneumonia cases in Wuhan in the Hubei Province [1]. Shortly after, a novel beta coronavirus—which is now designated as *severe acute respiratory syndrome coronavirus 2* (SARS-CoV-2) [2]—was discovered in the bronchoalveolar lavage fluid of a patient with pneumonia [1,3]. This enveloped virus has a single-stranded positive sense ribonucleic acid (RNA) genome of approximately 30 kilobases. Several surface proteins, known as the spike (S), envelope (E), and membrane (M) proteins, are embedded in a lipid bilayer. These proteins mediate the viral entry (S; S1 unit: binding to host receptor angiotensin-converting enzyme 2 via its receptor binding domain (RBD) and S2 unit: membrane fusion) and are also responsible for viral fusion (M), morphogenesis (E), assembly (E and M), and budding (M). The RNA is associated with the nucleocapsid (N) protein. The latter plays a role in the viral replication and transcription cycle [4,5,6,7].

SARS-CoV-2 emerged globally. As of 24 September 2020, the World Health Organization reported 31,664,104 SARS-CoV-2 infections and 972,221 deaths worldwide. So far, 278,070 cases and 10,982 deaths have been registered for Germany (Robert Koch-Institute, data of 24 September 2020).

The diagnosis of acute coronavirus-induced disease 2019 (COVID-19) requires the demonstration of SARS-CoV-2 RNA in respiratory samples by real-time reverse transcription polymerase-chain reaction (real-time RT-PCR). The specificity of the SARS-CoV-2 real-time RT-PCR has been reported up to 100% [8], while sensitivity under clinical conditions seems to be lower and was roughly estimated with 70% [9].

More recently, the measurement of the immune response against SARS-CoV-2 came into the focus of clinical diagnostics, particularly by the detection of virus-specific antibodies. The use of SARS-CoV-2 antibody tests could clarify the etiology of the disease in patients who present late, after two weeks from the onset of symptoms. These tests can also demonstrate the viral spread in the community and may even identify individuals who are potentially protected from reinfection by neutralizing antibodies [8].

It is believed that the majority of antibodies are raised against the abundant N protein, while antibodies directed against the S protein are considered more specific and correlate with the neutralizing capacity [7,8]. Earlier works on the antibody response in SARS (2002/2003) patients showed a significantly higher sensitivity of tests based on the N protein as the antigen [10,11]. The diagnostic value of SARS-CoV-2 antibody tests, however, may be limited due to their potential cross-reactivity with other human coronaviruses. So far, the duration of the acquired immunity after a SARS-CoV-2 infection is unclear. However, based on experience with reinfections from other human coronaviruses, it remains yet open if the immunity is long-lasting [12]. Several studies reported a rapid decay of SARS-CoV-2 immunoglobulin G (IgG) in asymptomatic individuals [13] and mild COVID-19 cases [14] but, more surprisingly, also in hospitalized patients presenting with the full clinical spectrum of COVID-19 [5,15]. Very recently, differences in the kinetics of N- or S-specific IgG came into the focus of research [16]. While a decline in the IgG directed against the N protein was evident, the response to the S protein or its RBD was found to be more stable [16,17] and was associated with the presence of virus-neutralizing antibodies [17]. To the best of our knowledge, clinically validated assays for the specific detection of IgG directed against the SARS-CoV-2 surface proteins E and M are not yet available. However, it would make sense to investigate such antibody responses as well, since they can also correlate with virus-neutralizing capacities [4].

In the following, we present data on the diagnostic performance of eight commercially available SARS-CoV-2 IgG or total SARS-CoV-2 antibody tests. Variations in their diagnostic sensitivity and specificity were observed. In terms of their performance, two IgG assays using the N or S proteins of SARS-CoV-2 were selected for an analysis of follow-up sera to obtain data on the kinetics of IgG. The development of IgG avidities and the kinetics of SARS-CoV-2-neutralizing antibodies were also examined over time.

## 2. Materials and Methods

The ethics committee of the Medical Faculty of Kiel University (Kiel, Germany) approved the setting of this study (reference number D467/20).

### 2.1. Reference Samples and Tests Chosen for Evaluation of Immunoassay Performance

Thirty-seven serum samples were obtained from 26 outpatient patients with a PCR-confirmed SARS-CoV-2 infection. Blood was drawn between four and 60 days (median 19 days) after a positive real-time RT-PCR result in which parts of the SARS-CoV-2 E or N genes [18] were detected in oropharyngeal swabs with a cycle threshold ≤35 (median 26.8). Serum was obtained from the blood and stored at −20 °C until tested. These sera are likely to contain SARS-CoV-2 IgG/total antibodies and, therefore, were considered to determine the serological assay sensitivities (as previously suggested by [19]). This variant of the sensitivity calculation only took into account whether a SARS-CoV-2-infected patient was reactive at all, regardless of when this reactivity was developed (reference method 1).

A plaque reduction neutralization assay (PRNT) using a SARS-CoV-2 isolate designated as Kiel M16502/2020 and Vero cells (order no. 605372, CLS Cell Lines Service GmbH, Eppelheim, Germany) was included as a further reference for the calculation of the sensitivities. All sera classified as reactive in the PRNT were considered. This also covered follow-up sera (reference method 2). Testing was done under biosafety level 3 conditions and was in accordance to previous reports [20,21] with minor modifications. One to two days before infection, 1.0 × 10^5^ cells were seeded into each well of 48-well plates. These were then incubated under standard conditions until the cells became confluent. Directly prior to the PRNT, patient sera were heat-inactivated at 56 °C for 30 min and then diluted from 1:10 to 1:1280 in cell culture medium consisting of Dulbecco’s modified Eagle’s medium (DMEM) (Bio&SELL GmbH, Feucht, Germany) supplemented with 3.7 g/L NaHCO_3_, 4.5 g/L glucose, 2 mM L-glutamine, and 1% (*v*/*v*) Penicillin/Streptomycin/Fungi-Mix (Bio&SELL). A serum dilution series was made by mixing 50 µL of each dilution step with 50 µL virus suspension containing 100 plaque-forming units, followed by an incubation for 1 h at 37 °C. The cells were washed with phosphate-buffered saline (Bio&SELL), inoculated with 100 μL of these virus serum dilutions, and incubated for one hour at room temperature on a rocking shaker. Then, 100 µL of the cell culture medium supplemented with 20% (*v*/*v*) fetal calf serum (FCS) was added to each well to achieve a FCS concentration of 10% (*v*/*v*). After four to five days, cells were fixed with 4% (*w*/*v*) paraformaldehyde in phosphate-buffered saline and stained with an aqueous solution of 0.1% (*w*/*v*) crystal violet and 20% (*v*/*v*) methanol. The stained plates were photo-documented. All dilution steps were tested in quadruplicates, and plaque formation was compared to an untreated cell control and a virus control. A serum dilution >1:10, which prevented plaque formation in at least 50% of the wells compared to the virus control, was classified as reactive and probably as protective. The reciprocal titers were used to visualize the kinetics. If an exact titer could not be given, a geometric mean value was calculated from the two adjacent titers.

For the calculation of assay specificities, 100 archived sera collected during the summer 2018 (N = 50) and during winter 2018/2019 (N = 50) were used. None of these sera were expected to contain SARS-CoV-2 antibodies. The inclusion of such pre-pandemic sera for the demonstration of specificity was previously suggested [19].

### 2.2. Investigated SARS-CoV-2 Antibody Tests

Seven SARS-CoV-2 IgG assays (Abbott SARS-CoV-2 IgG, Wiesbaden, Germany; DiaSorin Liaison^®^ SARS-CoV-2 S1/2 IgG, DiaSorin, Dietzenbach, Germany; Epitope EDI™ Novel Coronavirus COVID-19 IgG ELISA Kit, Epitope Diagnostics, San Diego, CA, USA; Euroimmun Anti-SARS-CoV-2 ELISA (IgG), EUROIMMUN AG, Lübeck, Germany; Mikrogen *recom*Well SARS-CoV-2 IgG, Mikrogen GmbH, Neuried, Germany; SARS-CoV-2 ViraChip^®^ IgG Test, Viramed Biotech AG, Planegg, Germany; and SERION ELISA agile SARS-COV-2 IgG, Institute Virion-Serion GmbH, Würzburg, Germany), as well as one total SARS-CoV-2 antibody test (Roche Elecsys Anti-SARS-CoV-2, Roche Diagnostics, Mannheim, Germany), were included in this study. The recombinant antigens of these tests cover the N protein (Abbott, Epitope, Mikrogen, Roche) and the entire S protein (Virion-Serion), as well as its S1 domain alone (Euroimmun) or together with the S2 domain (DiaSorin). The SARS-CoV-2 ViraChip^®^ IgG test kit uses the purified viral surface antigens S1 and S2 and the N antigen, which are all presented separately at a defined position on a nitrocellulose film (Table S1).

All tests were conducted strictly following the recommendations of the manufacturers on an Architect or Alinity machine (Abbott), a Liaison XL (DiaSorin), or a Cobas e 411 (Roche) or, for the assays of Epitope, Euroimmun, Mikrogen Viramed, and Virion-Serion, on the BEP 2000 system (Siemens Healthcare GmbH, Erlangen, Germany), respectively. The ViraChip^®^ test was then evaluated automatically by help of a ViraChip^®^ reader and the ViraChip^®^ Software.

All borderline/gray zone results were counted as positive. Furthermore, raw data from seven assays were converted into relative indices according to the decision limits set by the manufacturer. Thereby, a signal (S)/cut-off (CO) value of <1 was valued as negative and ≥1 as positive, which corresponds to a previous study [22]. Results of the SARS-CoV-2 ViraChip^®^ IgG could not be converted into relative indices and were only given qualitatively as negative or positive. The proportion of correctly identified samples per test relative to the chosen reference method was calculated and given as assay accuracy.

The convalescent sera were also tested with and without avidity reagent (contains urea in denaturating concentrations) in two versions of an IgG line assay (blot v1: Mikrogen *recom*Line Coronavirus IgG (Avidität), prototype and blot v2: Mikrogen *recom*Line SARS-CoV-2 IgG (Avidität)/RUO). The first version is based on the N protein of the human coronaviruses (HCoVs) 229E, HKU1, NL63, and OC43, which are used separately as antigens, and on the N protein of the “classic” SARS-CoV from 2002/2003 and SARS-CoV-2. The second improved version still uses the N proteins of the aforementioned HCoVs and of SARS-CoV-2 but has been expanded to include the S1 antigen and its RBD. A Dynablot Plus system was used to process all samples. Blots were then evaluated automatically with a BLOT*rix* reader and the *recom*Scan software (all from Mikrogen).

### 2.3. Kinetics of SARS-CoV-2 IgG Indices, IgG Avidities, and Neutralizing Antibody Titers

The SARS-CoV-2-IgG course was examined in 11 of the 26 SARS-CoV-2-infected patients over a period of up to 165 days after the first positive PCR result. For this purpose, the N- and S-specific IgG assays were selected, which showed the optimal performance depending on sensitivity, specificity, and accuracy. The sera were also tested in the improved IgG line assay, including the determination of the avidity. The course of the virus-neutralizing titers was examined in the PRNT (including the retesting of samples from the validation part of this study in order to show the assays’ reproducibility). An attempt was made to investigate all samples of a patient in one test run. Follow-up samples from five routine patients, including four family members from a SARS-CoV-2-infected case, were also examined.

## 3. Results

### 3.1. Performance of Eight SARS-CoV-2 IgG or Total Antibody Tests

**Figure 1:**
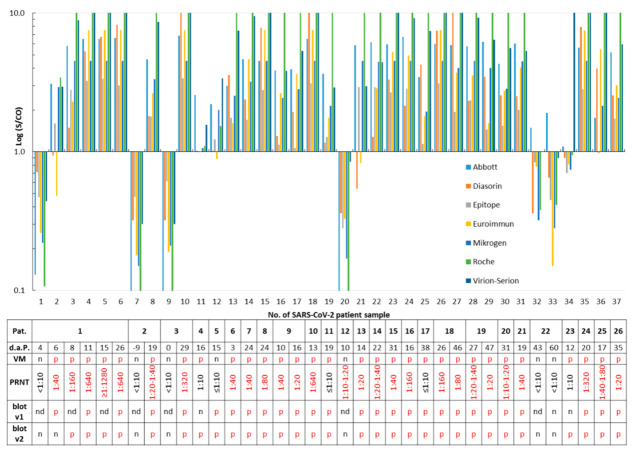
Detection of *severe acute respiratory syndrome coronavirus 2* (SARS-CoV-2) immunoglobulin G (IgG)/total antibodies in PCR-confirmed cases. Serum samples (N = 37) obtained from 26 SARS-CoV-2-infected patients (Pat.) were tested. A signal (S)/cut-off (CO) value ≥1 is considered as positive for SARS-CoV-2 IgG/total antibodies. The time in days after PCR (d.a.P.) and sample submission, as well as results of the ViraChip^®^ IgG test (Viramed, VM), of the plaque reduction neutralization assay (PRNT, Figure S1), and of both *recom*Line assays (blot v1 and v2) are listed in the table. p, positive; n, negative; and nd, not determined. For better clarity, positive test results are printed in red in the table.

Thirty-seven samples taken from patients with a PCR-confirmed SARS-CoV-2 infection were analyzed with eight SARS-CoV-2 IgG or total antibody assays. Samples #1, #7, and #9 were taken nine days before to four days after PCR and were all found to be free of SARS-CoV-2 antibodies by the assays. These samples were not considered for calculation of the sensitivity but demonstrated seroconversion (Figure 1). Out of the remaining 34 samples, only one serum (#20; Figure 1), which was obtained from patient no. 12 ten days after a positive RT-PCR, tested negative for SARS-CoV-2 IgG/total antibodies in the eight assays. However, this sample exhibited a PRNT 1:10–1:20. All other samples were reactive in at least one assay (Figure 1). Twenty (76.9%) patients developed virus-neutralizing antibodies, as shown by a PRNT >1:10 (Figure 1 and Figure S1). With respect to the SARS-CoV-2 PCR (reference 1) or the PRNT results (reference 2), assay sensitivities ranged from 80.8% to 96.3% (Table 1).

**Figure 2:**
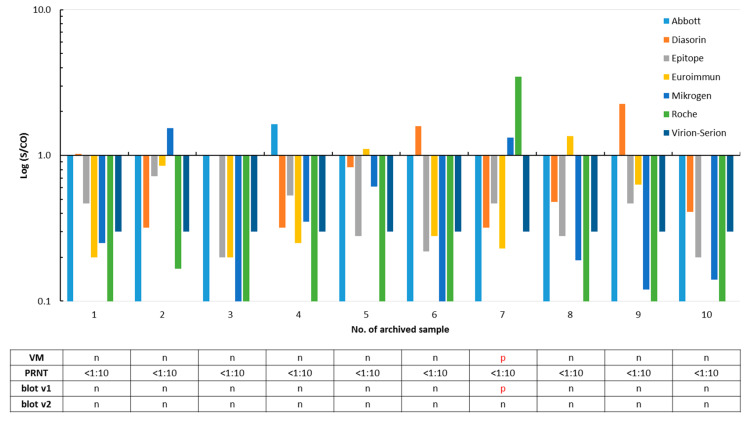
False positivity of SARS-CoV-2 IgG/total antibody tests in ten out of 100 archived sera collected in the summer 2018 and winter 2018/2019. A signal (S)/cut-off (CO) value ≥1 is considered as positive for SARS-CoV-2 IgG/total antibodies. Results of the ViraChip^®^ IgG test (Viramed, VM), of the plaque reduction neutralization assay (PRNT, Figure S1), and of both *recom*Line assays (blot v1 and v2) are listed in the table. p, positive and n, negative. For better clarity, positive test results are printed in red in the table.

Specificity was calculated using 100 archived samples that should not contain SARS-CoV-2 antibodies. A total of ten sera were reactive in one or more assays. None contained SARS-CoV-2 neutralizing antibodies (Figure 2 and Figure S1). The residual 90 stored samples were not tested in the PRNT, as they were found to be free of SARS-CoV-2 IgG/total antibodies in all eight assays. Thus, assay specificities ranged from 96.0% to 100%, and accuracies varied from 93.7% to 99.2% (Table 1).

**Figure 3:**
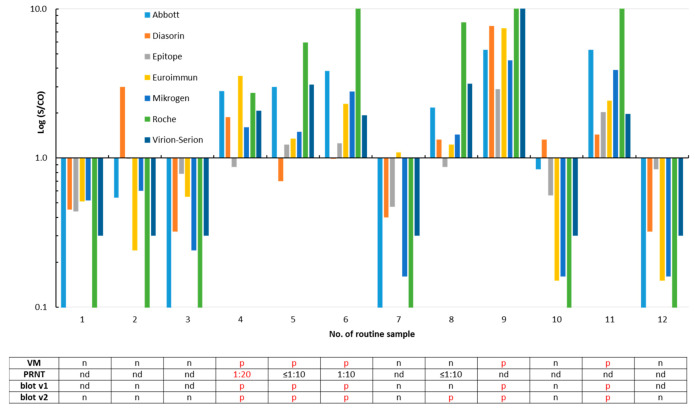
Detection of SARS-CoV-2 IgG/total antibodies in twelve routine samples. A signal (S)/cut-off (CO) value ≥1 is considered as positive for SARS-CoV-2 IgG/total antibodies. The samples #4 to #6 were obtained from family members of the SARS-CoV-2-infected patient no. 14 (sample #22 in Figure 1). Results of the plaque reduction neutralization test (PRNT, Figure S2), the ViraChip^®^ IgG test (Viramed, VM), and of both *recom*Line assays (blot v1 and v2) are listed in the table. p, positive; n, negative; and nd, not determined. For better clarity, positive test results are printed in red in the table.

Twelve routine samples obtained from individuals who were interested in their SARS-CoV-2 antibody status were re-evaluated by all eight assays (Figure 3). Six of them—including three family members of the SARS-CoV-2-infected patient no. 14 (Figure 1)—were classified SARS-CoV-2 IgG/total antibody-positive by the majority of the tests. Isolated reactivity was observed in three sera (Figure 3).

Twenty-four (92.3%) of the 26 SARS-CoV-2 infected patients were reactive in one or both versions of the IgG *recom*Line assay; the test results differed only in three sera (Figure 1). One out of the ten archived sera that were reactive in at least one of the SARS-CoV-2 IgG/total antibody tests was also classified as SARS-CoV-2 IgG-positive by the prototype immunoblot (v1) but was negative in the improved version 2. The underlying sample #7 was collected in the winter 2018/2019 and was found to be reactive for SARS-CoV-2 IgG/total antibodies by the Mikrogen (S/CO 1.33), Roche (S/CO 3.46), and Viramed (N antigen 125 ViraChip^®^ Units) assays in parallel (Figure 2). The twelve routine samples were analyzed by both versions of the blot (v1: nine samples and v2: all samples), and results of the immunoassays were largely confirmed (Figure 3).

### 3.2. Kinetics of SARS-CoV-2 IgG, Development of IgG Avidities, and Neutralizing Antibodies

**Figure 4:**
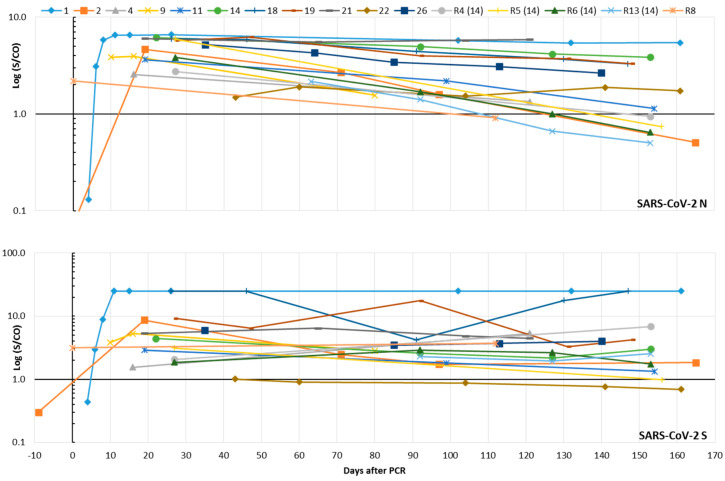
Time course of SARS-CoV-2 IgG in eleven SARS-CoV-2-infected patients and five routine (R) patients (including four family members of SARS-CoV-2 patient no. 14) who were interested in their SARS-CoV-2 antibody status. A signal (S)/cut-off (CO) value ≥1 is considered as positive for SARS-CoV-2 IgG. The results of the Abbott assay (N protein as the epitope) can be found in the upper part of the figure; those of the Virion-Serion assay (S protein as the epitope) are given below. Results from the validation part of this study were included.

In the validation part of our study, the Abbott assay, which is based on the SARS-CoV-2 N as the epitope, and the Virion-Serion assay, which uses S as the antigen, proved to be the most sensitive and specific assays (Table 1). Therefore, both were selected to study the kinetics of SARS-CoV-2 IgG in eleven patients and five individuals (including four family members of patient no. 14) from routine diagnostics. The median of the last sample submission was 153 days after the PCR, the range between 80 and 165 days. Results from the validation part of this study were also considered. N-specific IgG antibodies were detectable in all persons. Two groups of individuals were evident: one group of six patients showed relatively stable IgG levels over time, while the other showed a short-term decrease. Accordingly, the indices of six individuals fell below the limit of positivity during the observation period. A stable S-specific IgG response was demonstrated in almost all individuals (Figure 4). Only two subjects (patient no. 22 and routine no. 5) showed a drop in IgG below the limit of positivity. The routine sample no. 5 still possessed isolated reactivity against the N antigen in the improved line assay (data not shown). In the latter test, three patients developed SARS-CoV-2-specific IgG of high avidity after 65–130 days, which was confirmed in consecutive samples. The pattern of reactivity differed slightly: Two patients developed IgG of high avidity against the RBD and S1 epitopes, while the third showed isolated reactivity against the N antigen (Figure S3).

**Figure 5:**
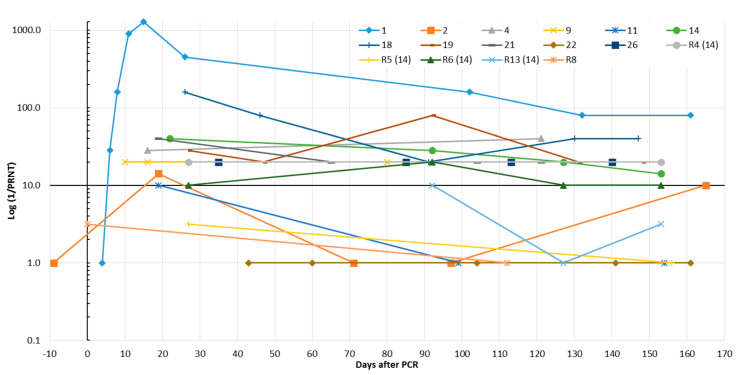
Time course of SARS-CoV-2-neutralizing antibodies in eleven SARS-CoV-2-infected patients and five routine (R) patients (including four family members of SARS-CoV-2 patient no. 14) who were interested in their SARS-CoV-2 antibody status. Results were obtained by the plaque reduction neutralization assay (PRNT, Figure S2). The reciprocal titer was calculated. If a clear titer could not be read, the geometric mean was given from the reciprocal values of the two neighboring titers.

Presence of SARS-CoV-2-neutralizing antibodies over several months was demonstrated in eight (72.7%) SARS-CoV-2 patients and a family member of patient no. 14 (R4). However, only one of them developed a stable titer >1:40 (Figure 5 and Figure S2). Retests of sera that were already included in the validation part of this study showed an excellent reproducibility of the PRNT (R^2^ = 0.96, Figure S4).

## 4. Discussion

In a rather short period, various assays for the detection of SARS-CoV-2 antibodies were introduced to the market. A meta-analysis of 38 studies on the performance of different format SARS-CoV-2 antibody tests mainly manufactured from Chinese companies was reported recently [23]. Furthermore, studies on the sensitivity and specificity of SARS-CoV-2 IgG tests, including ELISAs from the Epitope, Euroimmun, and Mikrogen companies, were published previously [20,21,22,24]. A recent prospective study examined the sensitivity of seven antibody tests from Abbott, Euroimmun, Mediagnost, Novatec, Virotech, Roche, and Siemens companies but did not provide data on their specificities or on the correlation of the assay results to the presence of SARS-CoV-2-neutralizing antibodies [25].

Here, we analyzed the diagnostic performance of eight commercially available tests, all of which have been released within the past few months. These were compared to identify appropriate tests to study the kinetics of N- or S-specific SARS-CoV-2 IgG or total antibodies in outpatients with a previous SARS-CoV-2 infection. To our knowledge, such a comprehensive study has not been conducted before. Three assays (Abbott, DiaSorin, and Roche) were applied in a random-access manner, which reduced the needed hands-on-time markedly. Convalescent sera from 26 SARS-CoV-2-infected patients were used to investigate the test sensitivities. These samples were characterized by a previous positive SARS-CoV-2 PCR result of the donors (reference method 1) and may possess SARS-CoV-2-neutralizing capacities (>1:10), as demonstrated in a laboratory-developed PRNT (reference method 2). Furthermore, two versions of an immunoblot, including the determination of SARS-CoV-2 IgG avidities, were applied.

With respect to the previous positive SARS-CoV-2 PCR result considered as the reference of first choice, following a recent suggestion [19], assay sensitivities were found to be relatively high and varied from 80.8% to 96.2% (Table 1). This finding is in accordance to other studies [21,22,24,25,26] and to a meta-analysis [23]. Six (23.1%; five, 19.2%, if the positive retesting result of the initial serum from patient 4 is taken into account; for details, see below) of the 26 SARS-CoV-2-infected patients did not develop SARS-CoV-2-neutralizing antibodies, as demonstrated by a PRNT ≤1:10. Among them were two individuals who were only recognized as IgG-positive in the Abbott and Virion-Serion assays and were partially reactive in the ViraChip^®^ and the IgG immunoblots (Figure 1). This confirms a previous report of a high sensitivity of the Abbott IgG test [27]. The absence of neutralizing antibodies in several COVID-19 patients was also reported by others [28] and may be associated with the mild course of the infection mainly observed in outpatient patients. However, since no clinical data were available to us, a severe underlying disease or a serious course of the SARS-CoV-2 infection cannot be ruled out in the individuals of our study collective. Recently, presence of neutralizing antibodies was found to be associated with protection against reinfection [29]. If the PRNT was chosen as another reference (Figure S1), sensitivities varied from 88.9% to 96.3% (Table 1), which is largely comparable to the results obtained with the first method. These sensitivities can be even higher, as retesting of the initial serum from patient 4 in the second part of our study revealed the presence of low-level neutralizing antibodies (1:10 vs. 1:20–1:40; Figure 5 and Figures S1 and S2). With respect to a sample from patient 1 taken six days after the PCR, two S-based assays failed to detect SARS-CoV-2 IgG, while all assays that used the N as an epitope were reactive (Figure 1). Thus, usage of the latter may lead to the detection of IgG/total antibodies at an earlier stage post-infection. Furthermore, a lack of reactivity in some assays using the S antigen does not necessarily mean that SARS-CoV-2-neutralizing antibodies are missing (Figure 1). Seroconversion was clearly demonstrated in three patients (Figure 1). The majority of SARS-CoV-2 IgG in sera from SARS-CoV-2-infected patients were verified by both versions of an immunoblot (Figure 1). The latter can be used to differentiate the antibody reactivities against individual SARS-CoV-2 antigens. The specificities of the SARS-CoV-2 IgG/total antibody tests are at comparatively high levels between 96.0% and 100.0%, as has also been reported by others [21,22,24,26]. Interestingly, one sample collected in the winter 2018/2019 was found to be reactive in the Mikrogen, Roche, and Viramed assays, as well as in the prototype immunoblot, but could not be confirmed by the PRNT. For all other 99 archived samples, a random pattern of rare, isolated reactivity was demonstrated, and none was confirmed as possessing SARS-CoV-2-neutralizing antibodies by PRNT (Figure 2 and Figure S1). Potential cross-reactivity with the HCoVs responsible for common colds may therefore not be a major problem. The analysis of the immunoblot results shows, however, that sera from SARS-CoV-2-infected patients may simultaneously react with the antigens from HCoVs 229E, HKU-1, NL63, OC43, and SARS-CoV-2 (Figure S3). Due to the currently estimated low prevalence of SARS-CoV-2 IgG in the overall German population, even high specificities of 96–98% would produce a relevant number of false positive results. Thus, such assays, which all show an accuracy over 93%, should be preferentially used for the testing of patients with a history of a probable infection. In the case of doubt, the implementation of the labor-intensive and time-consuming PRNT may be considered.

The kinetics of SARS-CoV-2 IgG was studied up to 165 days after positive SARS-CoV-2 PCR results by the analysis of consecutive samples from 11 patients and samples from five routine patients; four are family members of a SARS-CoV-2-infected patient. The results were obtained with two IgG assays based on the N (Abbott) or the entire S (Virion-Serion) antigens as the epitopes. Both tests turned out to be the most sensitive and specific assays in the validation part of our study (compare in Table 1). Thereby, SARS-CoV-2-specific antibodies were detectable in all individuals. While the N-based IgG assay revealed the existence of two groups of individuals, and one of them showed a rapid decrease of IgG indices within a few months, the IgG reactivity in the S protein-based assay was found to be more stable (Figure 4). This is in-line with a previous preprint [16] and could suggest that the latter test is better suited for epidemiological studies, especially when it comes to questions of prevalence. Accordingly, the majority of SARS-CoV-2-infected patients in our study developed neutralizing antibodies. The titers were stable during the observation period but were in a relatively low range between >1:10 and 1:80 (Figure 5). Only three patients showed conversion from low to high SARS-CoV-2 IgG avidity (Figure S3). The limited number of included SARS-CoV-2 patients, however, limits the informative value of the present study. The evaluability of the simple and reproducible PRNT technique applied here may be improved by overlaying the cells with cellulose or by using specific antibodies to detect the remaining viral antigens in the cells, as reported previously [7,20]. Furthermore, the application of a modified cytopathic effect inhibition assay [30] may be useful to facilitate standardization and quantification and will be valued in future studies. Several SARS-CoV-2-infected patients and routine patients in our study lost their SARS-CoV-2 (N)-specific IgG within a few months or could lose them soon. These individuals can theoretically be prone to reinfection, especially since the S-specific IgG and the neutralizing antibodies are stable but only present in low indices/titers. In a few patients, both were undetectable. However, this view does not take into account the booster effect that can be expected in the event of renewed virus contact. This would most likely lead to a rapid increase in antibody titers. Accordingly, vaccine strategies against SARS-CoV-2 may need boosting to maintain sufficient neutralizing antibody titers over a long time [5]. Waning antibody titers and reinfections are known from various HCoVs mainly responsible for common colds [5,12,31]. Recently a well-documented case of an asymptomatic SARS-CoV-2 reinfection was reported [32]. In our opinion, the importance of such rare events for the progression of the temporary pandemic appears to be minor. Nevertheless, a permanent presence of measurable SARS-CoV-2 IgG in the serum of all patients is unlikely. This is not surprising and has also been observed in patients after infections with SARS-CoV (2002/2003) or Middle East respiratory syndrome (MERS)-CoV [5]. It does not necessarily mean that these individuals will be susceptible to severe reinfection.

Future studies should now address the virus-specific antibody response on the mucous membranes, as well as the cellular immune response. For this, the development of valid routine test procedures is urgently required.

## Figures and Tables

**Table 1:** Sensitivities, specificities, and accuracies of eight *severe acute respiratory syndrome coronavirus 2* (SARS-CoV-2) immunoglobulin G (IgG) or total antibody assays calculated from the results of two reference tests. The highest values are shown in green; the lowest values are marked in red.

(%)	Sensitivity	Sensitivity	Specificity ^3^	Accuracy	Accuracy
Reference test	PCR ^1^	PRNT ^2^		PCR ^1^	PRNT ^2^
Assay\n	26	27	100	126	127
Abbott	96.2	96.3	99.0	98.4	98.4
Diasorin	84.6	88.9	96.0	93.7	94.5
Epitope	80.8	92.6	100.0	96.0	98.4
Euroimmun	80.8	88.9	97.0	93.7	95.3
Mikrogen	88.5	96.3	98.0	96.0	97.6
Roche	88.5	96.3	99.0	96.8	98.4
Viramed	88.5	96.3	99.0	96.8	98.4
Virion-Serion	96.2	96.3	100.0	99.2	99.2

^1^ SARS-CoV-2 RNA was previously detected in the PCR test. ^2^ SARS-CoV-2-neutralizing antibodies with a titer >1:10 were demonstrated in the plaque reduction neutralization test (PRNT). ^3^ 100 stored sera from 2018/2019.

## Supplementary Materials

The following are available online at https://www.mdpi.com/2076-2607/8/10/1572/s1. Table S1: Characteristics of the eight SARS-CoV-2 IgG/total antibody tests. Figure S1: Raw data from the plaque reduction neutralization test used as reference # 2 in the validation part of this study. Figure S2: Raw data from the plaque reduction neutralization test to investigate the kinetics of the virus-neutralizing antibodies. Figure S3: Development of high SARS-CoV-2 IgG avidities in three SARS-CoV-2 patients. Figure S4: Agreement of titers from two independent neutralization tests.
