# Supplementary material for: Kinetics of Nucleo- and Spike Protein-Specific Immunoglobulin G and of Virus-Neutralizing Antibodies after SARS-CoV-2 Infection
Source: Microorganisms. 2020 Oct 13;8(10):1572. doi: 10.3390/microorganisms8101572 (PMC7650537; doi:10.3390/microorganisms8101572)
Supplement: Supplementary file 1 [file microorganisms-08-01572-s001.zip › supplementary_material/Figure_S1_revised.pdf]

Figure S1: Raw data from the plaque reduction neutralization test used as reference #2 in the validation part of this study.

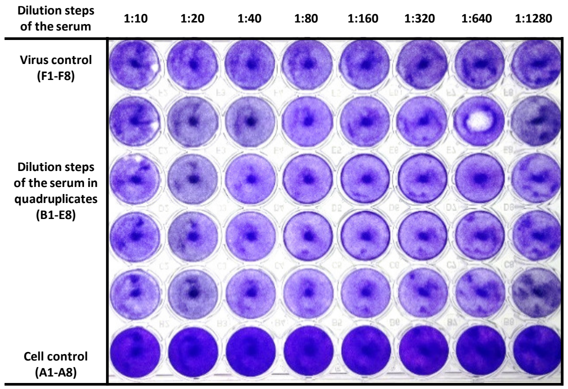

Archived serum (12641696, #3):
PRNT <1:10.

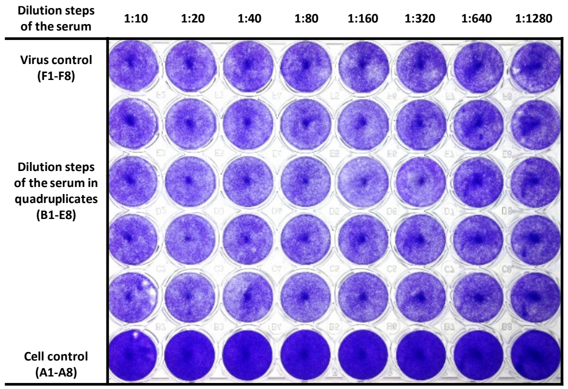

Archived serum (12700966, #8):
PRNT <1:10.

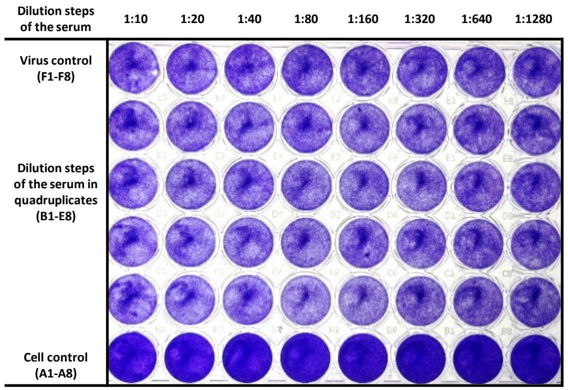

Archived serum (41354992, #1):
PRNT <1:10.

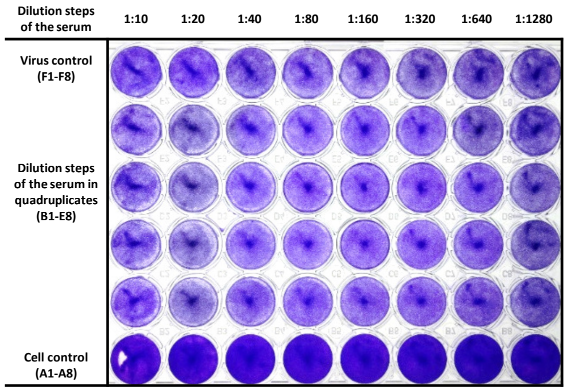

Archived serum (41379572, #2):
PRNT <1:10.

Figure S1: *continued.*

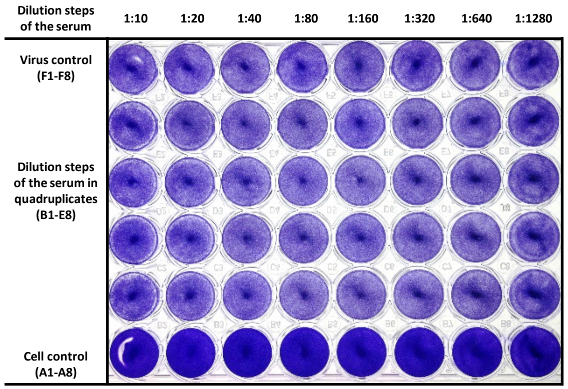

Archived serum (41413833, #5):
PRNT <1:10.

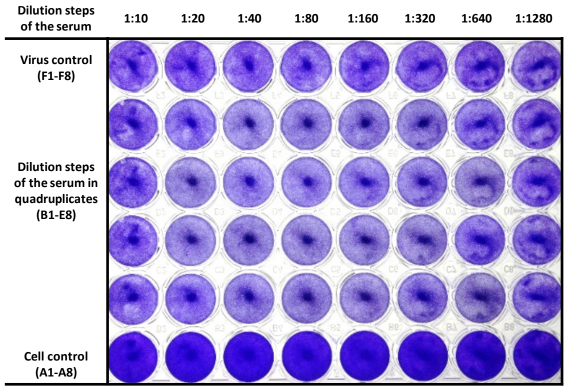

Archived serum (41418016, #4):
PRNT <1:10.

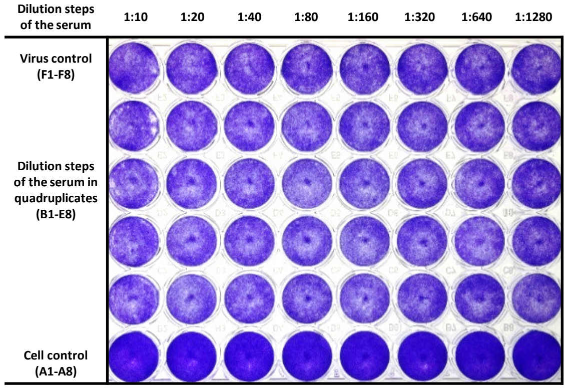

Archived serum (41533704, #6):
PRNT <1:10.

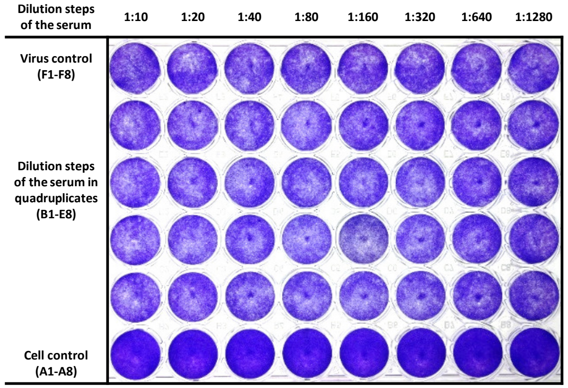

Archived serum (41543646, #7):
PRNT <1:10.

Figure S1: *continued.*

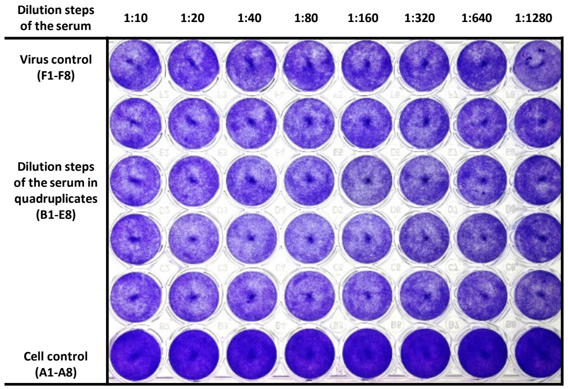

Archived serum (41572497, #9):
PRNT <1:10.

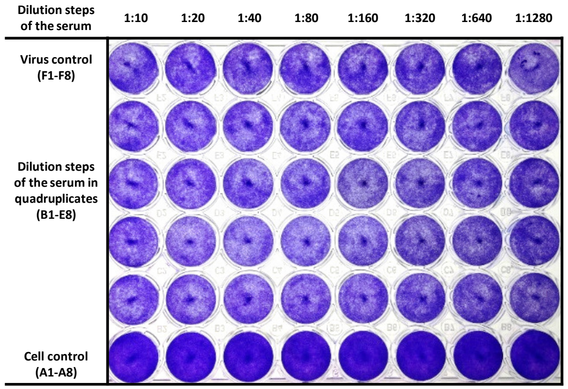

Archived serum (41580810, #10):
PRNT <1:10.

Figure S1: *continued.*

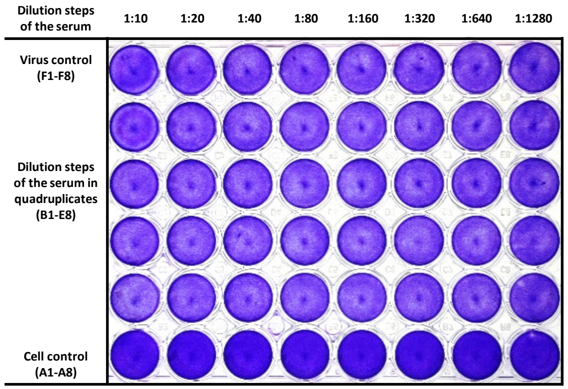

SARS-CoV-2 patient 1 (4 d.a.P.):
PRNT <1:10.

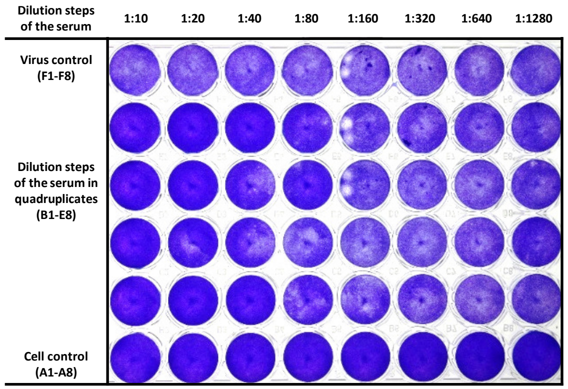

SARS-CoV-2 patient 1 (6 d.a.P.):
PRNT 1:40.

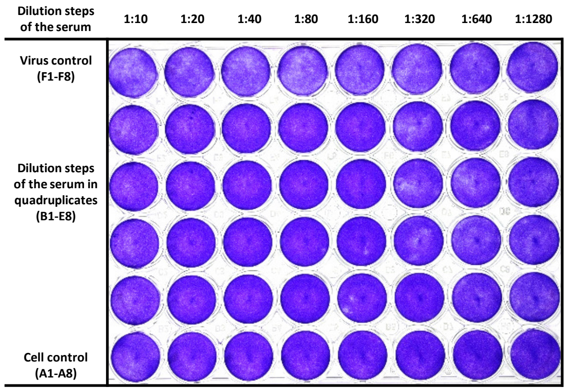

SARS-CoV-2 patient 1 (8 d.a.P.):
PRNT 1:160.

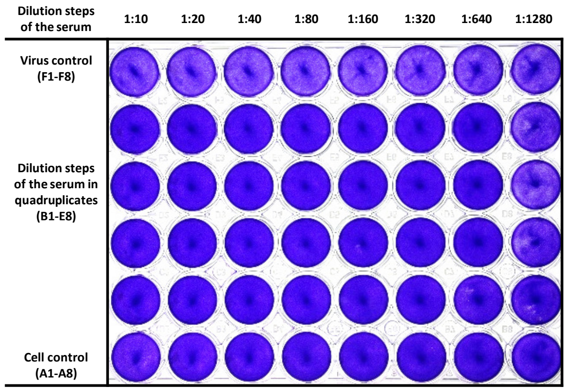

SARS-CoV-2 patient 1 (11 d.a.P.):
PRNT 1:640.

d.a.P., days after the PCR
d.b.P., days before the PCR

Figure S1: *continued.*

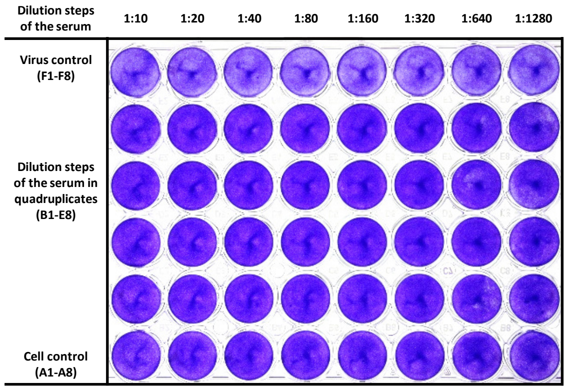

SARS-CoV-2 patient 1 (15 d.a.P.):
PRNT ≥1:1280.

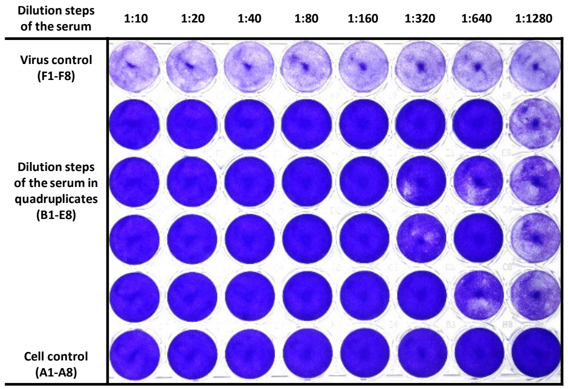

SARS-CoV-2 patient 1 (26 d.a.P.):
PRNT 1:640.

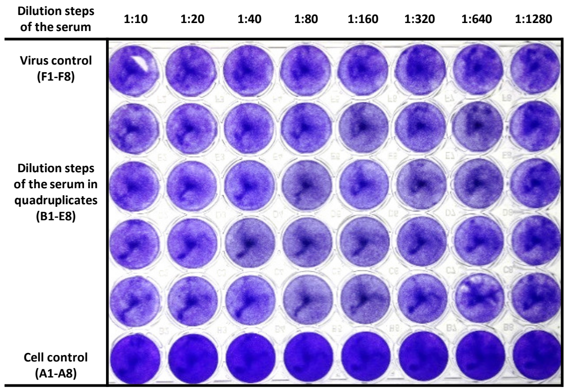

SARS-CoV-2 patient 2 (9 d.b.P.):
PRNT <1:10.

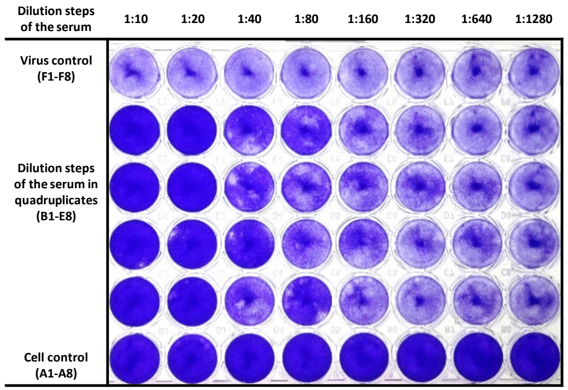

SARS-CoV-2 patient 2 (19 d.a.P.):
PRNT 1:20 - 1:40.

d.a.P., days after the PCR
d.b.P., days before the PCR

Figure S1: *continued.*

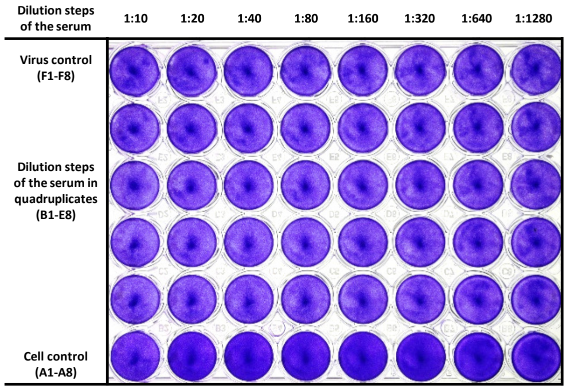

SARS-CoV-2 patient 3 (0 d.a.P.):
PRNT <1:10.

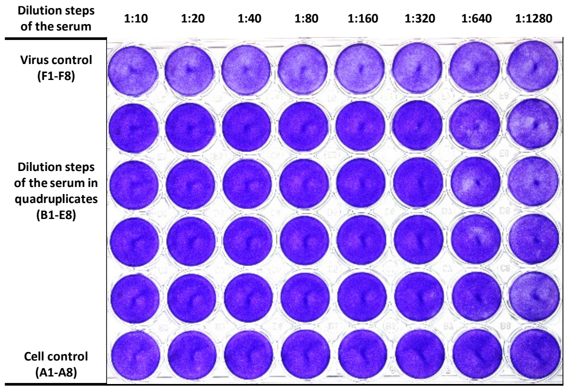

SARS-CoV-2 patient 3 (29 d.a.P.):
PRNT 1:320.

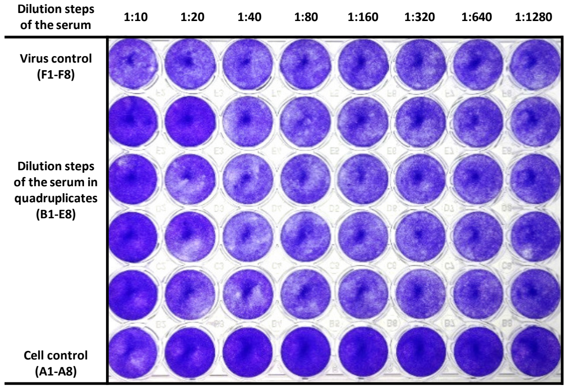

SARS-CoV-2 patient 4 (16 d.a.P.):
PRNT 1:10.

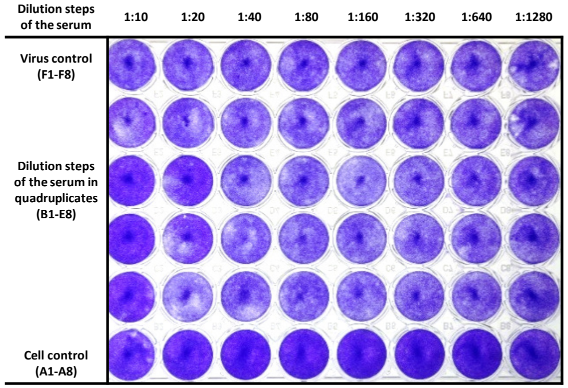

SARS-CoV-2 patient 5 (15 d.a.P.):
PRNT ≤ 1:10.

d.a.P., days after the PCR
d.b.P., days before the PCR

Figure S1: *continued.*

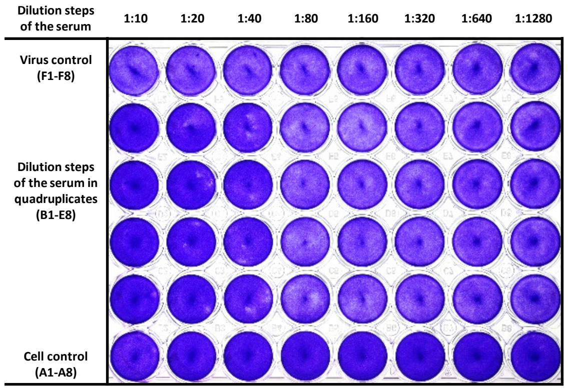

SARS-CoV-2 patient 6 (3* d.a.P.):
PRNT 1:40.
* The patient reported longstanding symptoms

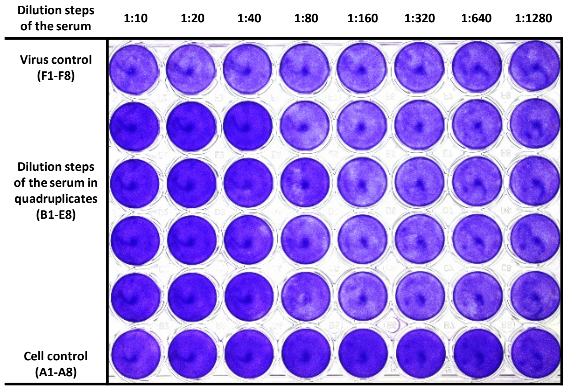

SARS-CoV-2 patient 7 (24 d.a.P.):
PRNT 1:40.

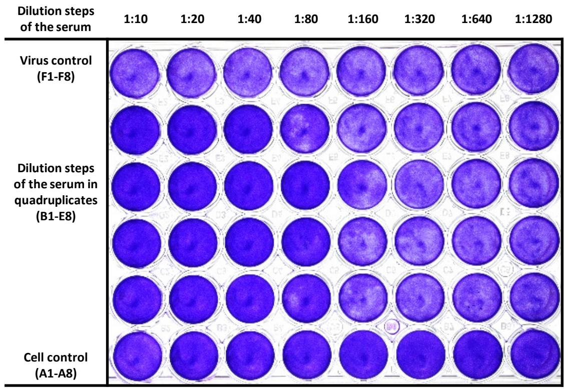

SARS-CoV-2 patient 8 (24 d.a.P.):
PRNT 1:80.

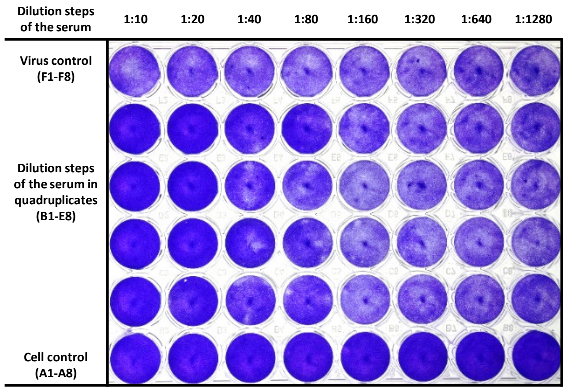

SARS-CoV-2 patient 9 (10 d.a.P.):
PRNT 1:40.

d.a.P., days after the PCR
d.b.P., days before the PCR

Figure S1: *continued.*

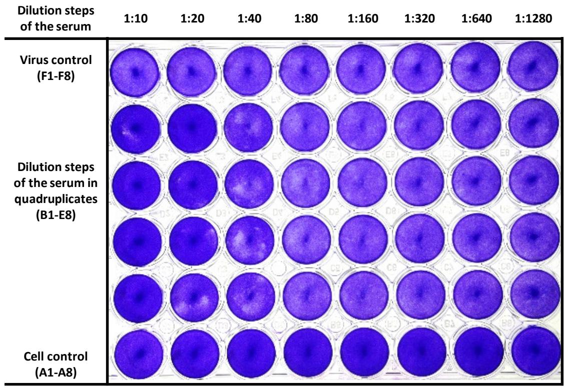

SARS-CoV-2 patient 9 (16 d.a.P.):
PRNT 1:20.

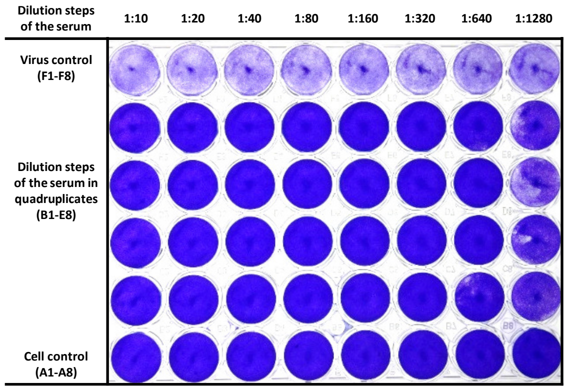

SARS-CoV-2 patient 10 (13 d.a.P.):
PRNT 1:640.

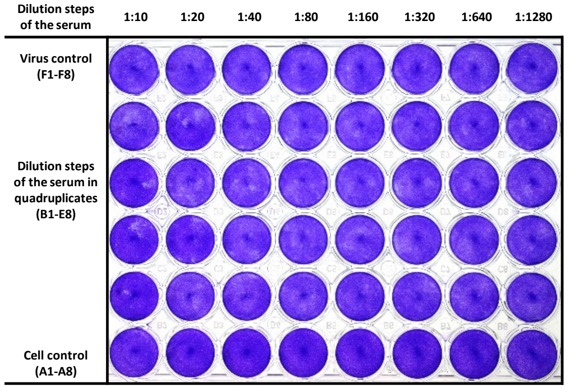

SARS-CoV-2 patient 11 (19 d.a.P.):
PRNT ≤ 1:10.

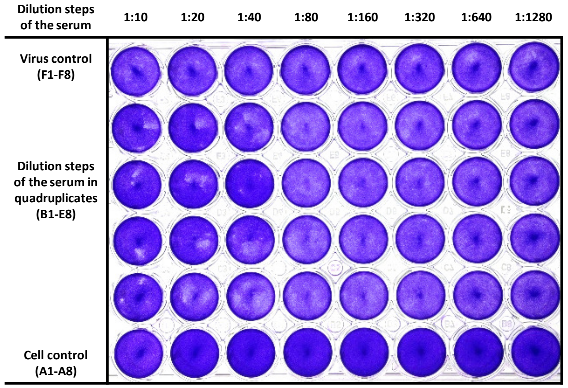

SARS-CoV-2 patient 12 (10 d.a.P.):
PRNT 1:10 - 1:20.

d.a.P., days after the PCR
d.b.P., days before the PCR

Figure S1: *continued.*

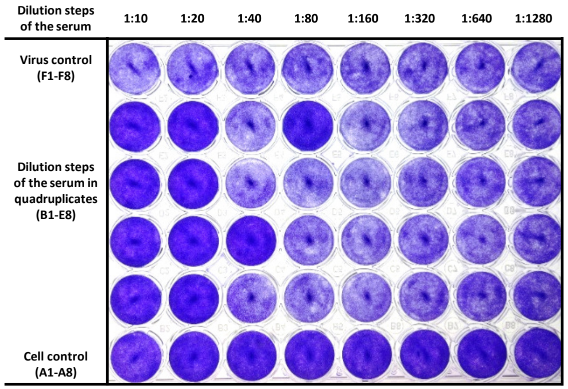

SARS-CoV-2 patient 13 (14 d.a.P.):
PRNT 1:20.

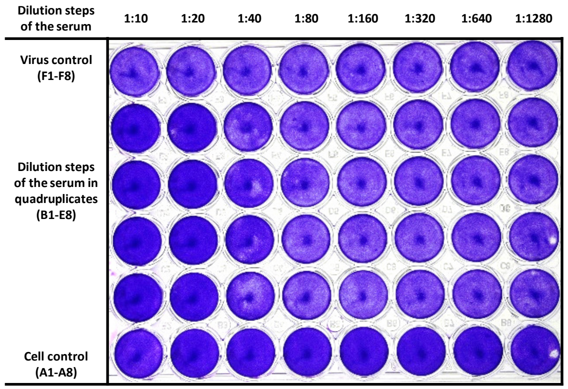

SARS-CoV-2 patient 14 (22 d.a.P.):
PRNT 1:20 - 1:40.

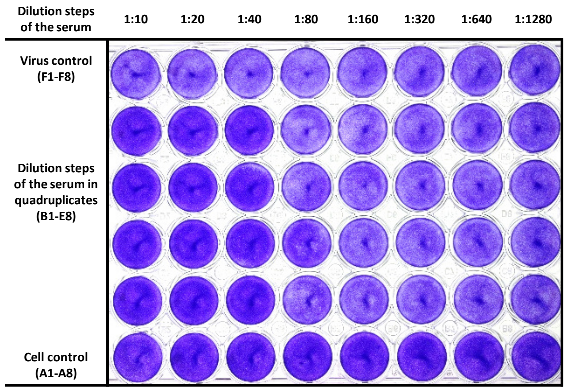

SARS-CoV-2 patient 15 (31 d.a.P.):
PRNT 1:40.

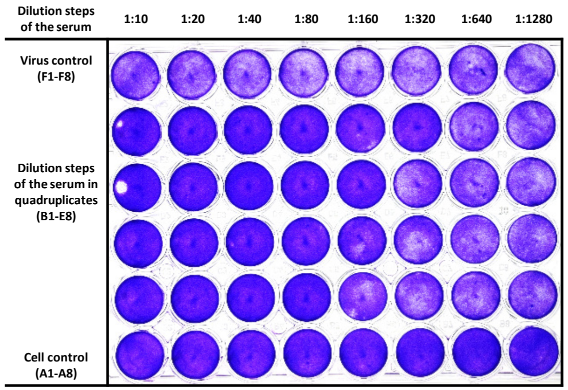

SARS-CoV-2 patient 16 (16 d.a.P.):
PRNT 1:160.

d.a.P., days after the PCR
d.b.P., days before the PCR

Figure S1: *continued.*

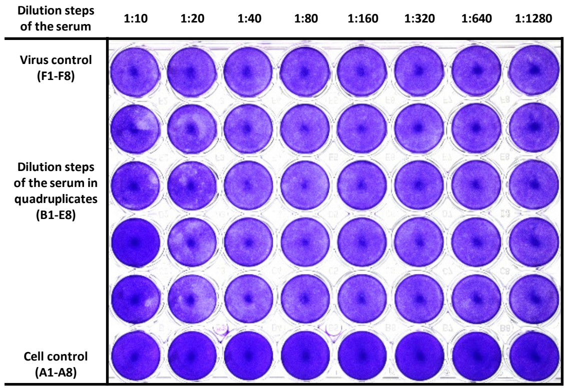

SARS-CoV-2 patient 17 (38 d.a.P.):
PRNT ≤ 1:10.

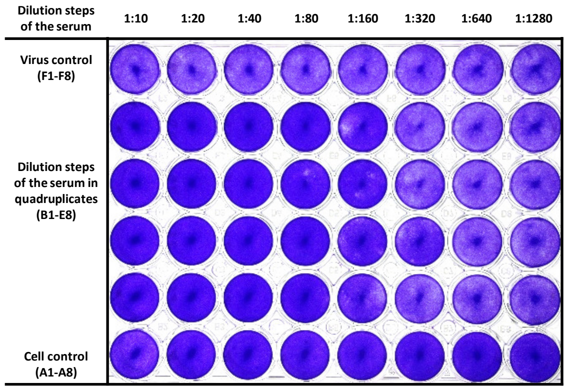

SARS-CoV-2 patient 18 (26 d.a.P.):
PRNT 1:160.

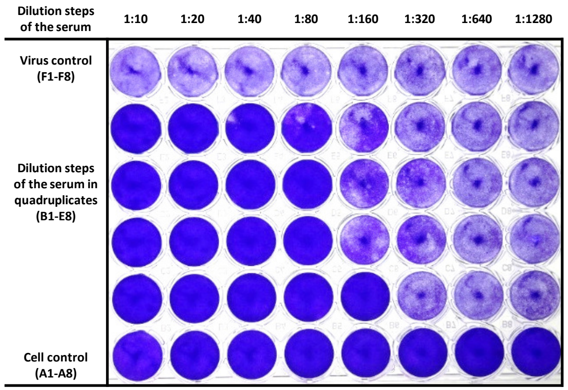

SARS-CoV-2 patient 18 (46 d.a.P.):
PRNT 1:80.

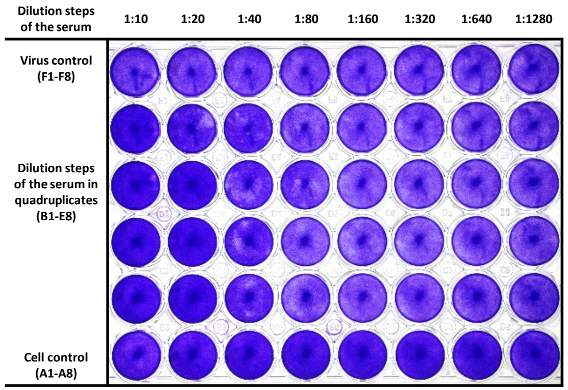

SARS-CoV-2 patient 19 (27 d.a.P.):
PRNT 1:20 - 1:40.

d.a.P., days after the PCR
d.b.P., days before the PCR

Figure S1: *continued.*

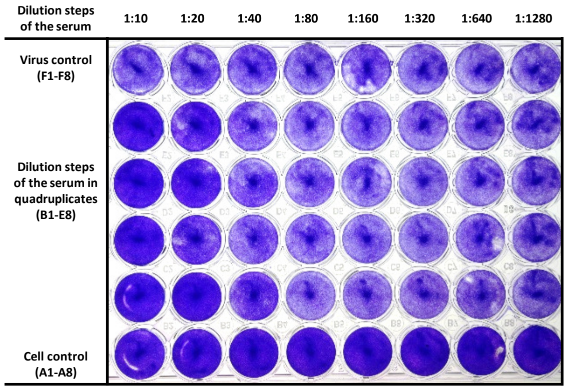

SARS-CoV-2 patient 19 (47 d.a.P.):
PRNT 1:20.

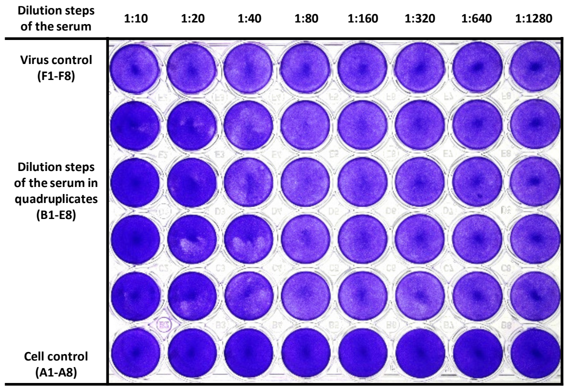

SARS-CoV-2 patient 20 (31 d.a.P.):
PRNT 1:10 - 1:20.

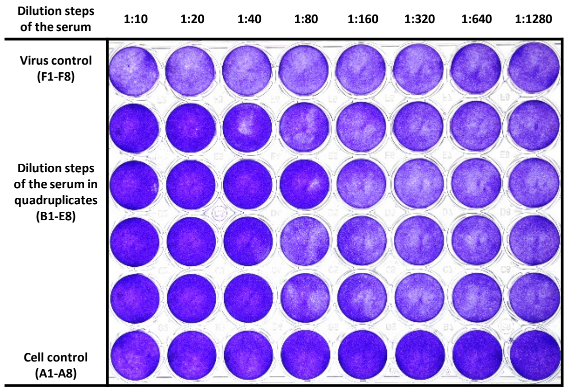

SARS-CoV-2 patient 21 (19 d.a.P.):
PRNT 1:40.

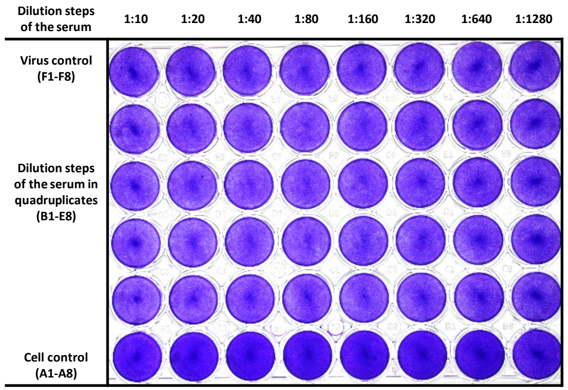

SARS-CoV-2 patient 22 (43 d.a.P.):
PRNT <1:10.

d.a.P., days after the PCR
d.b.P., days before the PCR

Figure S1: *continued.*

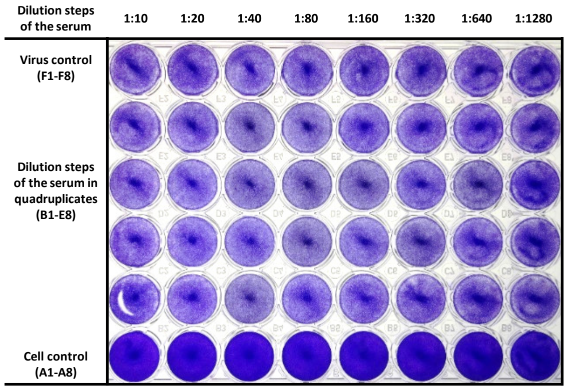

SARS-CoV-2 patient 22 (60 d.a.P.):
PRNT <1:10.

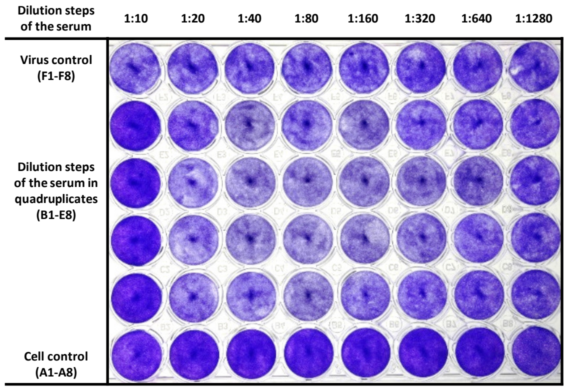

SARS-CoV-2 patient 23 (12 d.a.P.):
PRNT 1:10.

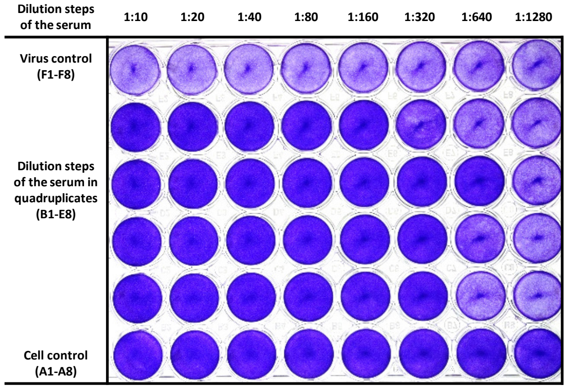

SARS-CoV-2 patient 24 (20 d.a.P.):
PRNT 1:320.

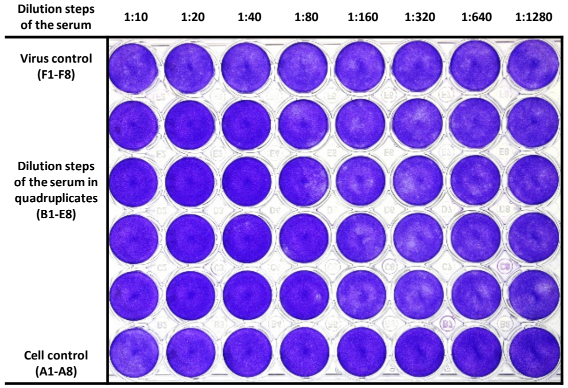

SARS-CoV-2 patient 25 (17 d.a.P.):
PRNT 1:40 - 1:80.

d.a.P., days after the PCR
d.b.P., days before the PCR

Figure S1: *continued.*

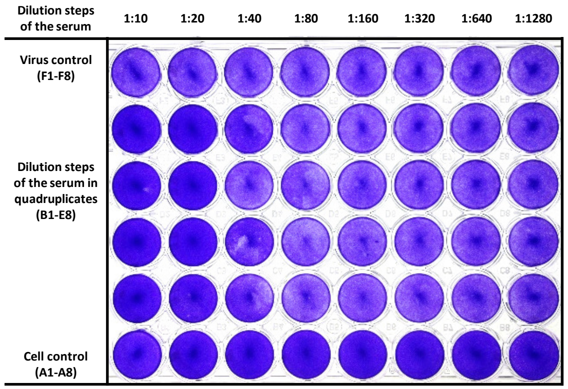

SARS-CoV-2 patient 26 (35 d.a.P.):
PRNT 1:20.

d.a.P., days after the PCR
d.b.P., days before the PCR
