# Supplementary material for: Kinetics of Nucleo- and Spike Protein-Specific Immunoglobulin G and of Virus-Neutralizing Antibodies after SARS-CoV-2 Infection
Source: Microorganisms. 2020 Oct 13;8(10):1572. doi: 10.3390/microorganisms8101572 (PMC7650537; doi:10.3390/microorganisms8101572)
Supplement: Supplementary file 1 [file microorganisms-08-01572-s001.zip › supplementary_material/Figure_S2_revised.pdf]

Figure S2: Raw data from the plaque reduction neutralization test to investigate the kinetics of virus-neutralizing antibodies.

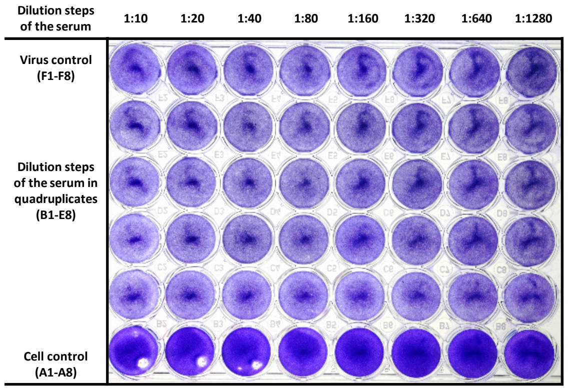

SARS-CoV-2 patient 1 (4 d.a.P.):
$PRNT_{50}$: < 1:10.

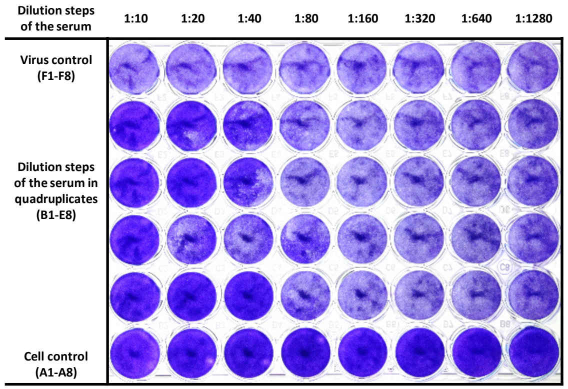

SARS-CoV-2 patient 1 (6 d.a.P.):
$PRNT_{50}$: 1:20 – 1:40.

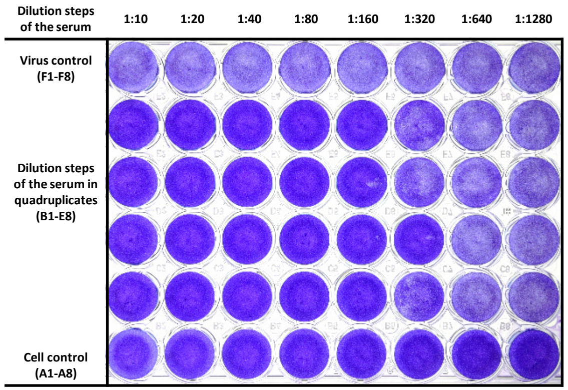

SARS-CoV-2 patient 1 (8 d.a.P.):
$PRNT_{50}$: 1:160.

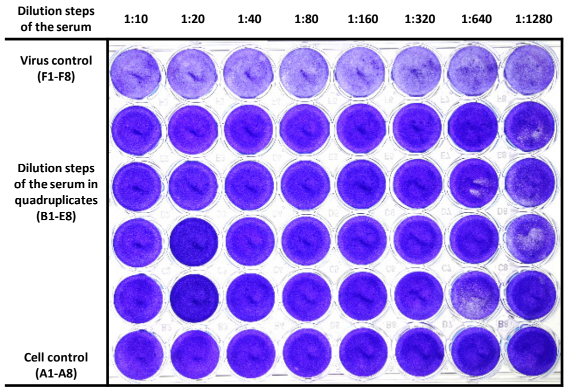

SARS-CoV-2 patient 1 (11 d.a.P.):
$PRNT_{50}$: 1:640 – 1:1280.

d.a.P., days after positive PCR test

Figure S2: *continued.*

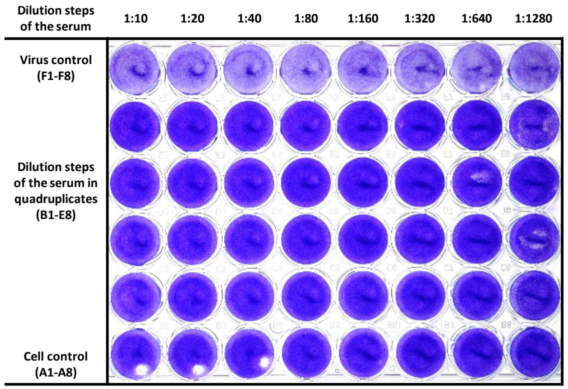

SARS-CoV-2 patient 1 (15 d.a.P.):
$PRNT_{50}$: ≥ 1:1280.

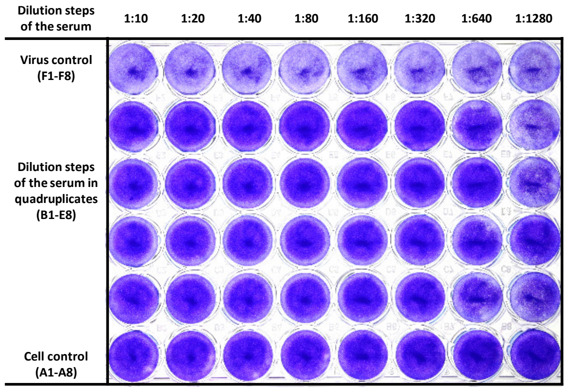

SARS-CoV-2 patient 1 (26 d.a.P.):
$PRNT_{50}$: 1:320 – 1:640.

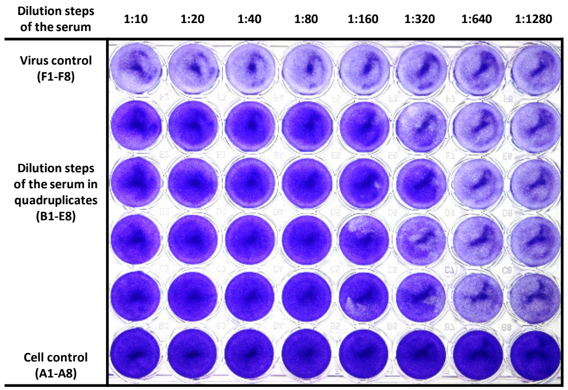

SARS-CoV-2 patient 1 (102 d.a.P.):
$PRNT_{50}$: 1:160.

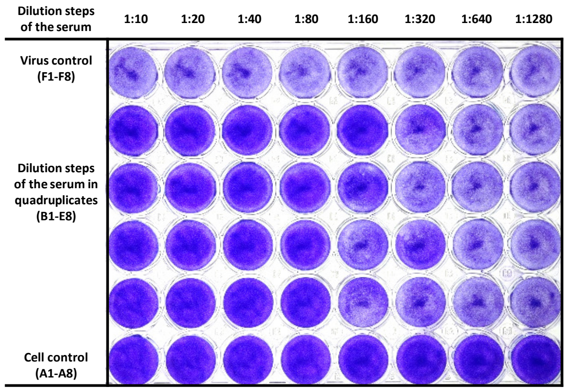

SARS-CoV-2 patient 1 (132 d.a.P.):
$PRNT_{50}$: 1:80.

d.a.P., days after positive PCR test

Figure S2: *continued.*

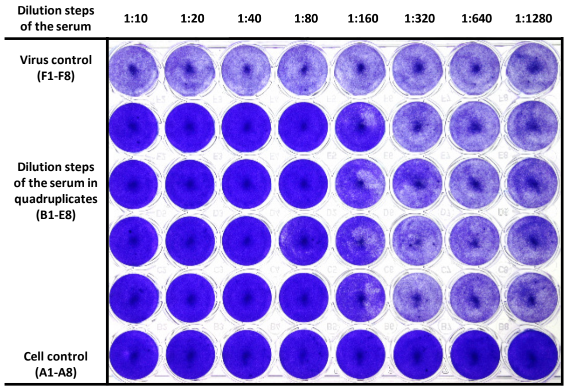

SARS-CoV-2 patient 1 (161 d.a.P.):
$PRNT_{50}$: 1:80.

d.a.P., days after positive PCR test

Figure S2: *continued.*

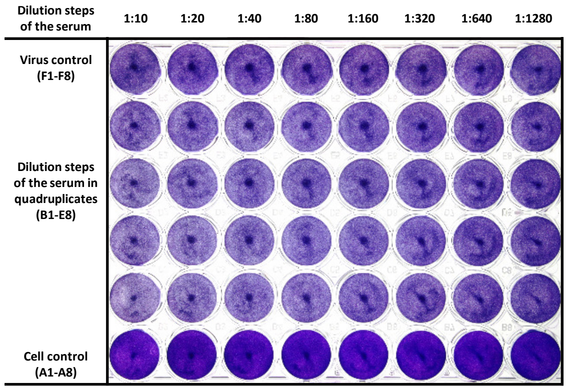

SARS-CoV-2 patient 2 (9 d.b.P.):
PRNT$_{50}$: < 1:10.

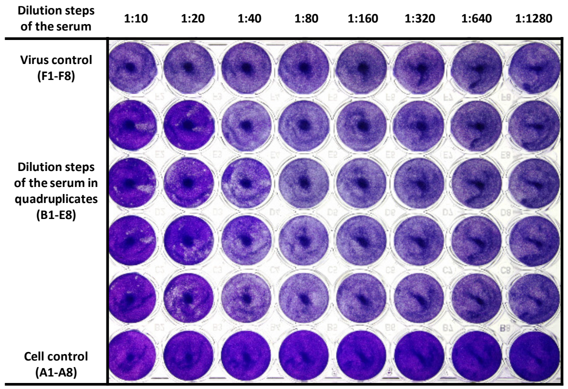

SARS-CoV-2 patient 2 (19 d.a.P.):
PRNT$_{50}$: 1:10 – 1:20.

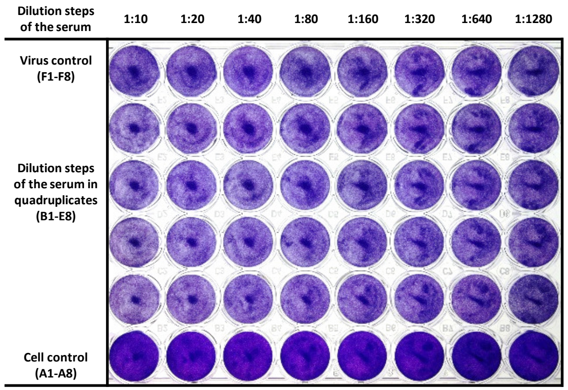

SARS-CoV-2 patient 2 (71 d.a.P.):
PRNT$_{50}$: < 1:10.

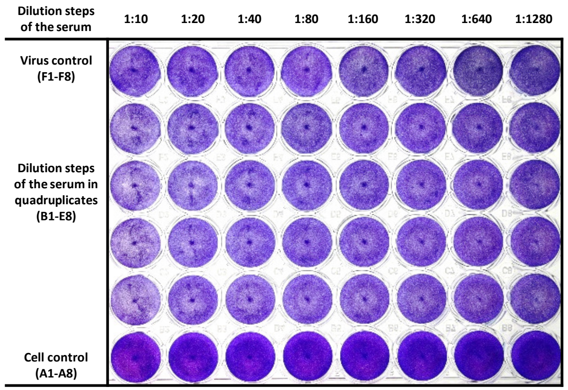

SARS-CoV-2 patient 2 (97 d.a.P.):
PRNT$_{50}$: < 1:10.

d.a.P., days after positive PCR test
d.b.P., days before the positive PCR test

Figure S2: *continued.*

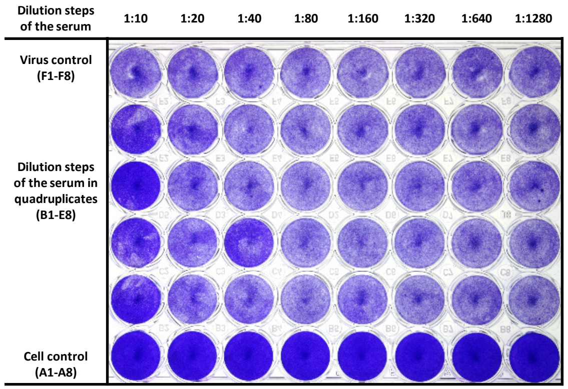

SARS-CoV-2 patient 2 (165 d.a.P.):
$PRNT_{50}$: 1:10.

d.a.P., days after positive PCR test

Figure S2: *continued.*

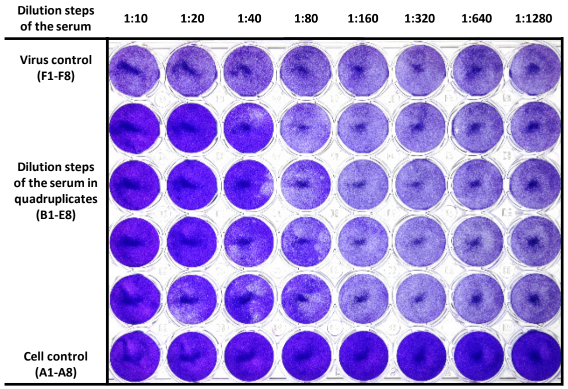

SARS-CoV-2 patient 4 (14 d.a.P.):
$PRNT_{50}$: 1:20 – 1:40.

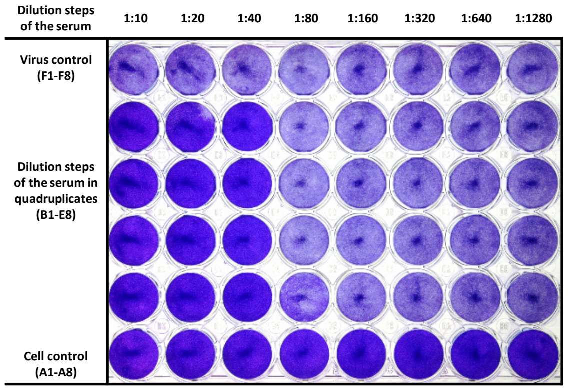

SARS-CoV-2 patient 4 (121 d.a.P.):
$PRNT_{50}$: 1:40.

d.a.P., days after positive PCR test

Figure S2: *continued.*

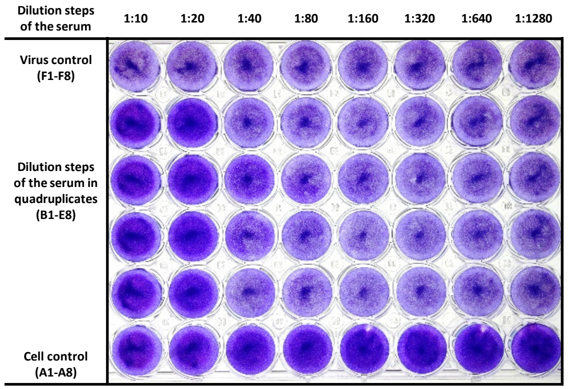

SARS-CoV-2 patient 9 (10 d.a.P.):
PRNT$_{50}$: 1:20.

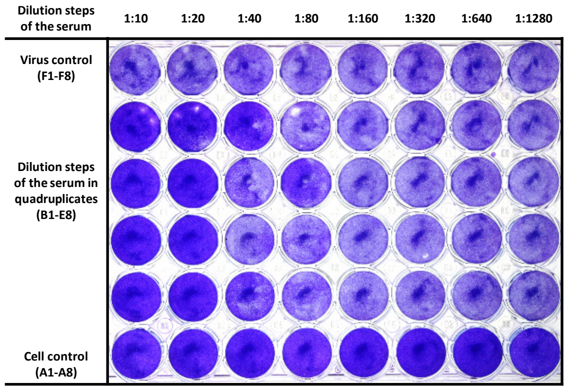

SARS-CoV-2 patient 9 (16 d.a.P.):
PRNT$_{50}$: 1:20.

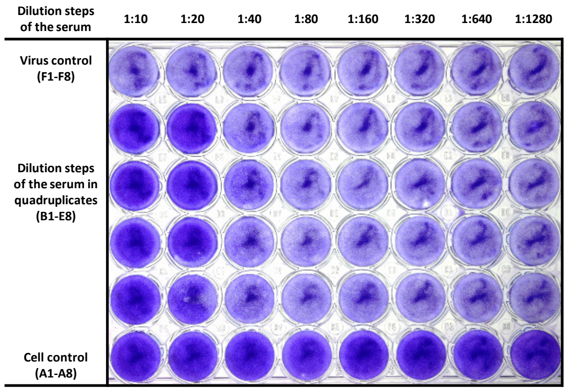

SARS-CoV-2 patient 9 (80 d.a.P.):
PRNT$_{50}$: 1:20.

d.a.P., days after positive PCR test

Figure S2: *continued.*

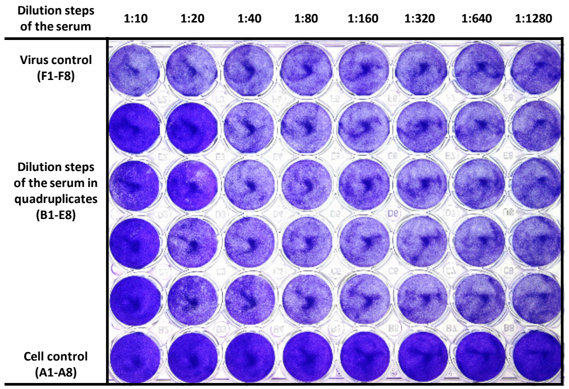

SARS-CoV-2 patient 11 (19 d.a.P.):
$PRNT_{50}$: 1:10.

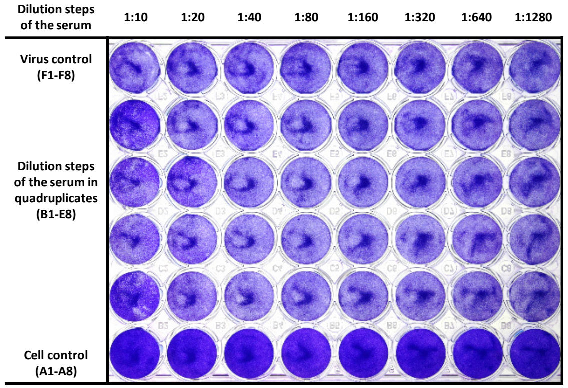

SARS-CoV-2 patient 11 (99 d.a.P.):
$PRNT_{50}$: < 1:10.

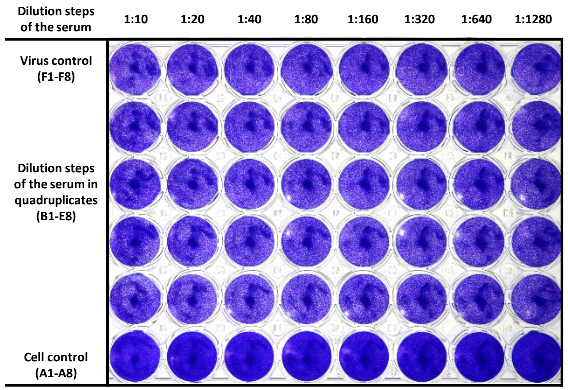

SARS-CoV-2 patient 11 (154 d.a.P.):
$PRNT_{50}$: < 1:10.

d.a.P., days after positive PCR test

Figure S2: *continued.*

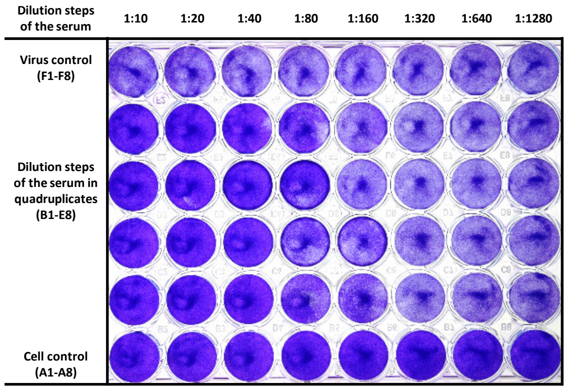

SARS-CoV-2 patient 14 (22 d.a.P.):
$PRNT_{50}$: 1:40.

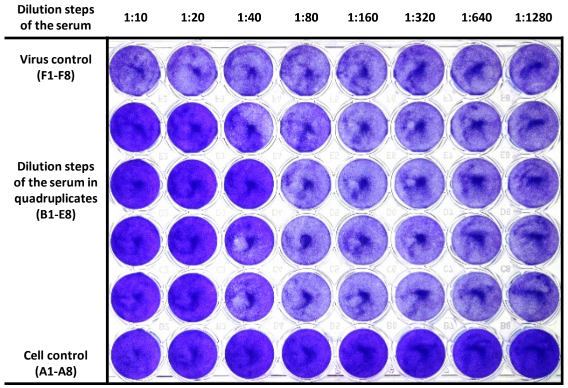

SARS-CoV-2 patient 14 (92 d.a.P.):
$PRNT_{50}$: 1:20 – 1:40.

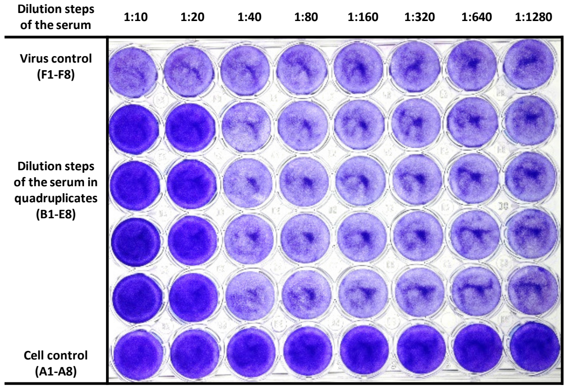

SARS-CoV-2 patient 14 (127 d.a.P.):
$PRNT_{50}$: 1:20.

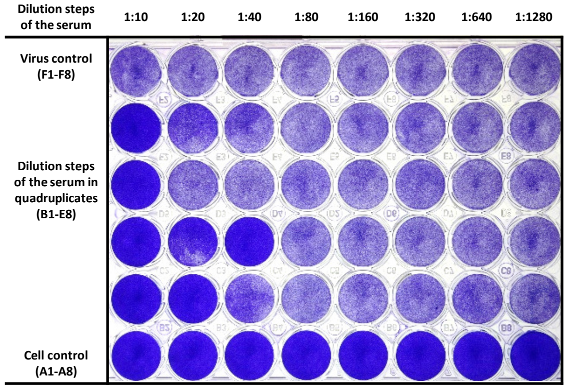

SARS-CoV-2 patient 14 (153 d.a.P.):
$PRNT_{50}$: 1:10 – 1:20.

d.a.P., days after positive PCR test

Figure S2: *continued.*

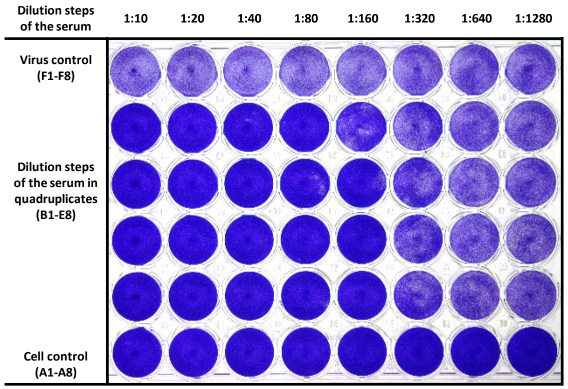

SARS-CoV-2 patient 18 (26 d.a.P.):
$PRNT_{50}$: 1:160.

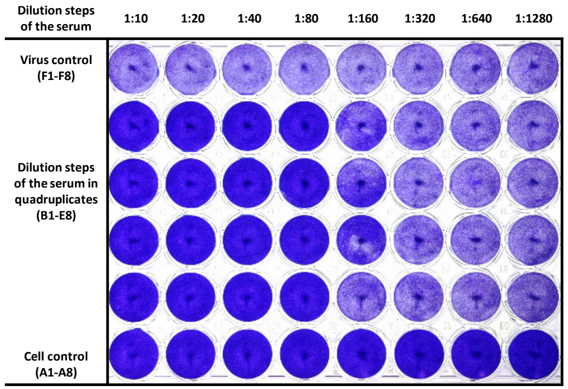

SARS-CoV-2 patient 18 (46 d.a.P.):
$PRNT_{50}$: 1:80.

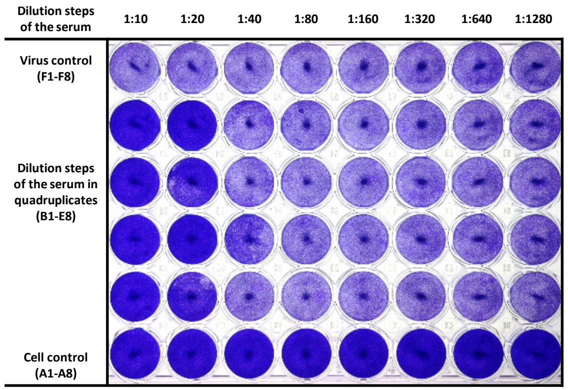

SARS-CoV-2 patient 18 (91 d.a.P.):
$PRNT_{50}$: 1:20.

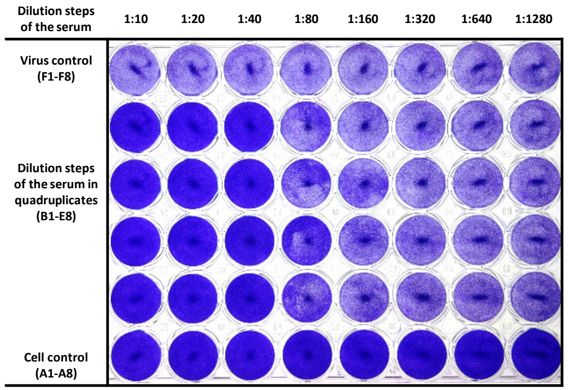

SARS-CoV-2 patient 18 (130 d.a.P.):
$PRNT_{50}$: 1:40.

d.a.P., days after positive PCR test

Figure S2: *continued.*

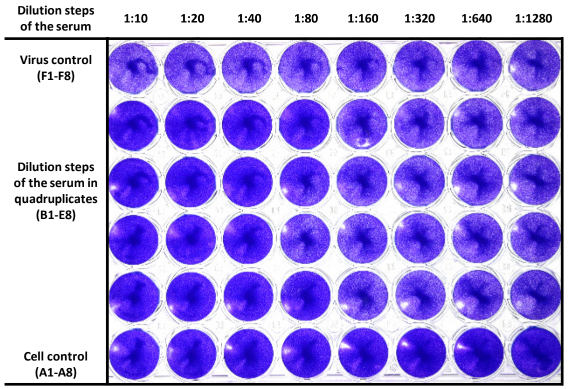

SARS-CoV-2 patient 18 (147 d.a.P.):
PRNT_{50}: 1:40.

d.a.P., days after positive PCR test

Figure S2: *continued.*

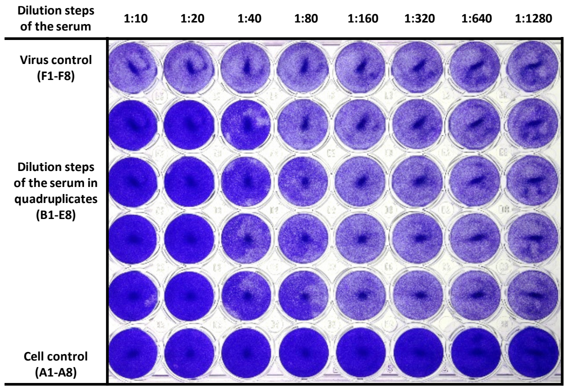

SARS-CoV-2 patient 19 (27 d.a.P.):
$PRNT_{50}$: 1:20 – 1:40.

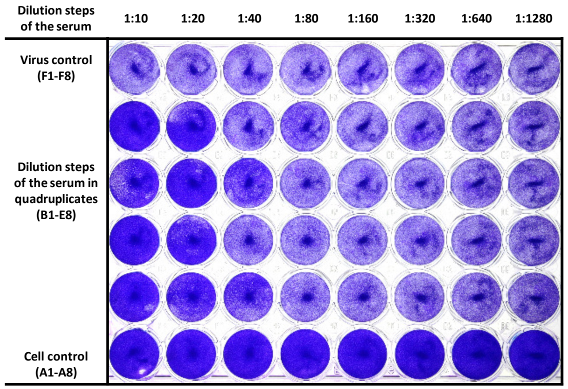

SARS-CoV-2 patient 19 (47 d.a.P.):
$PRNT_{50}$: 1:20.

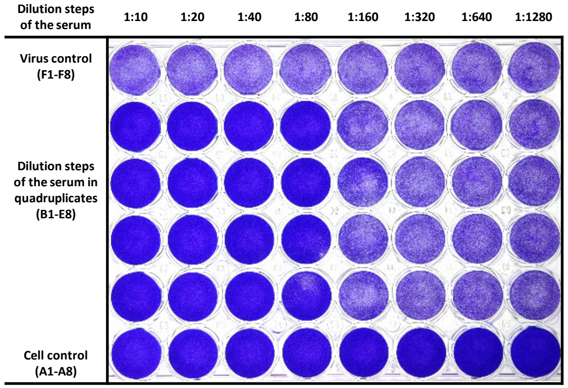

SARS-CoV-2 patient 19 (92 d.a.P.):
$PRNT_{50}$: 1:80.

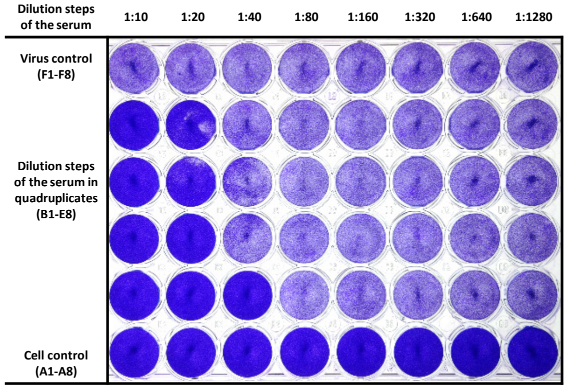

SARS-CoV-2 patient 19 (131 d.a.P.):
$PRNT_{50}$: 1:20.

d.a.P., days after positive PCR test

Figure S2: *continued.*

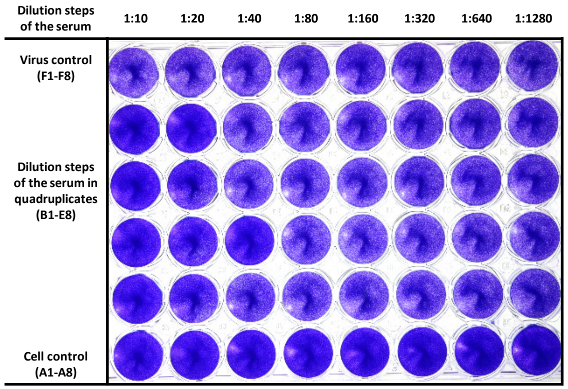

SARS-CoV-2 patient 19 (148 d.a.P.):
$PRNT_{50}$: 1:20.

d.a.P., days after positive PCR test

Figure S2: *continued.*

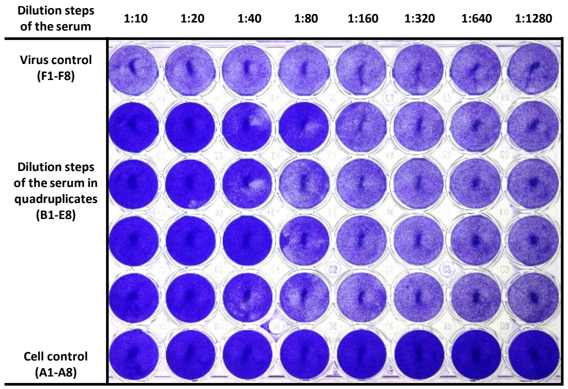

SARS-CoV-2 patient 21 (19 d.a.P.):
$PRNT_{50}$: 1:40.

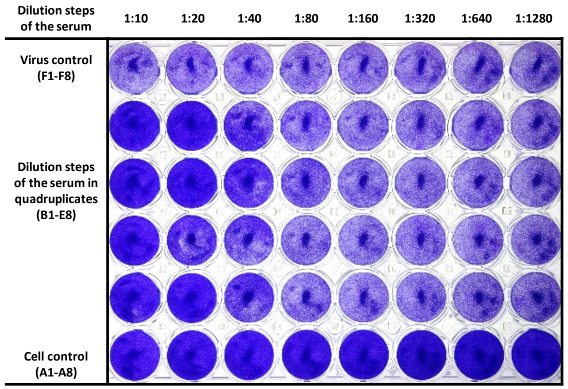

SARS-CoV-2 patient 21 (65 d.a.P.):
$PRNT_{50}$: 1:20.

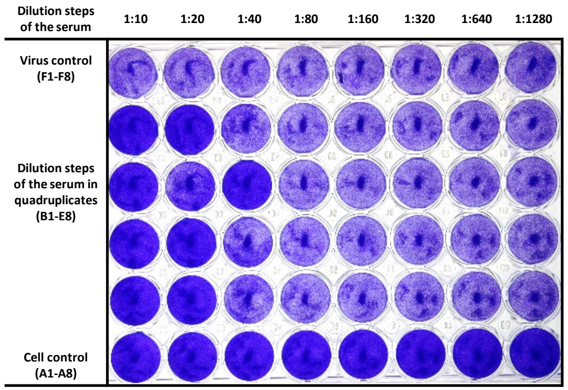

SARS-CoV-2 patient 21 (104 d.a.P.):
$PRNT_{50}$: 1:20.

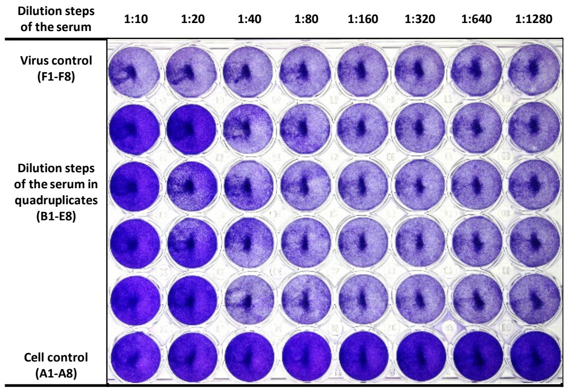

SARS-CoV-2 patient 21 (121 d.a.P.):
$PRNT_{50}$: 1:20.

d.a.P., days after positive PCR test

Figure S2: *continued.*

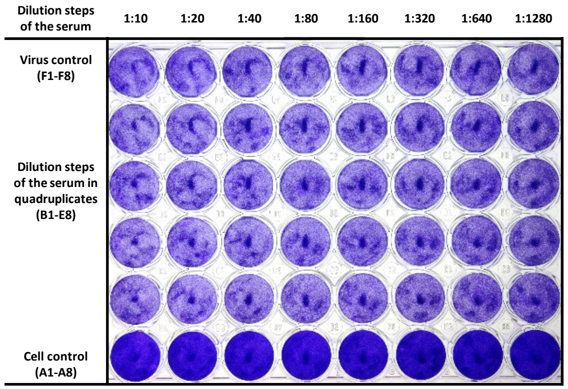

SARS-CoV-2 patient 22 (46 d.a.P.):
$PRNT_{50}$: < 1:10.

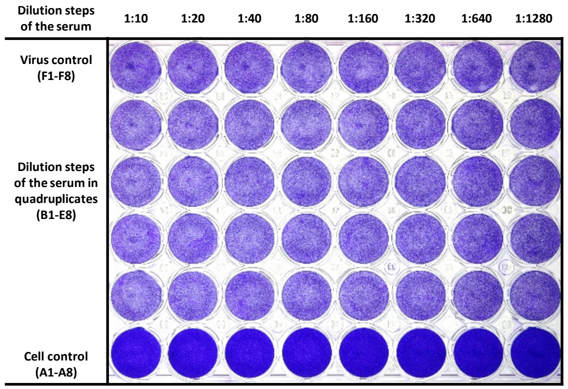

SARS-CoV-2 patient 22 (60 d.a.P.):
$PRNT_{50}$: < 1:10.

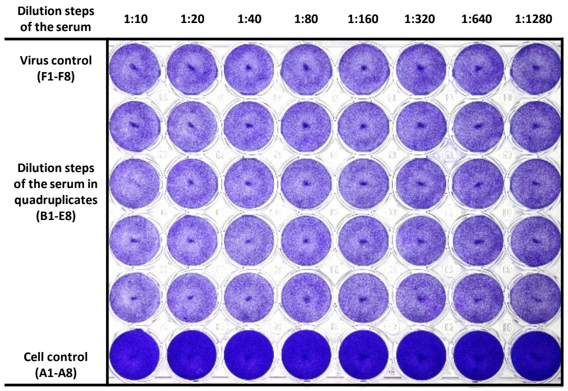

SARS-CoV-2 patient 22 (104 d.a.P.):
$PRNT_{50}$: < 1:10.

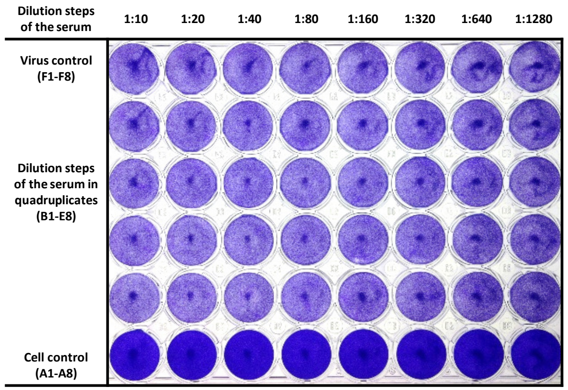

SARS-CoV-2 patient 22 (141 d.a.P.):
$PRNT_{50}$: < 1:10.

d.a.P., days after positive PCR test

Figure S2: *continued.*

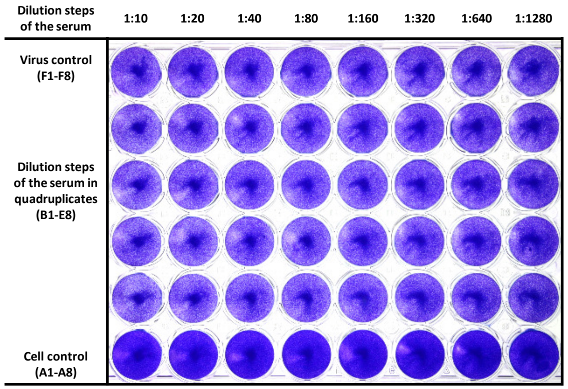

SARS-CoV-2 patient 22 (161 d.a.P.):
$PRNT_{50}$: < 1:10.

d.a.P., days after positive PCR test

Figure S2: *continued.*

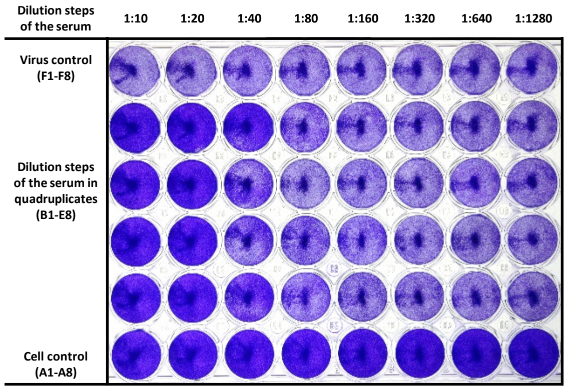

SARS-CoV-2 patient 26 (35 d.a.P.):
$PRNT_{50}$: 1:20.

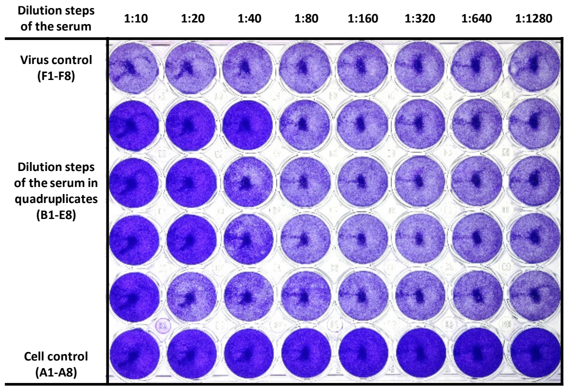

SARS-CoV-2 patient 26 (85 d.a.P.):
$PRNT_{50}$: 1:20.

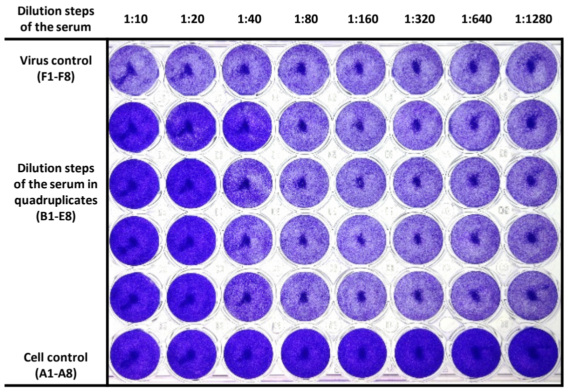

SARS-CoV-2 patient 26 (113 d.a.P.):
$PRNT_{50}$: 1:20.

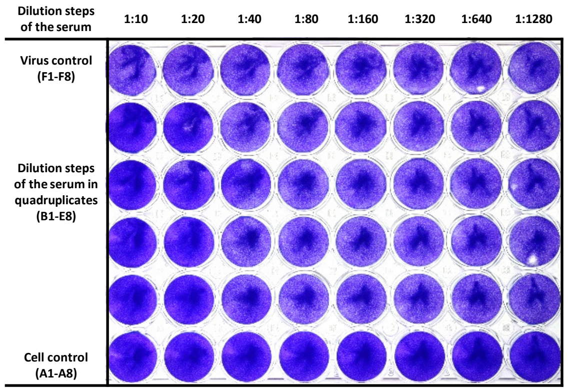

SARS-CoV-2 patient 26 (140 d.a.P.):
$PRNT_{50}$: 1:20.

d.a.P., days after positive PCR test

Figure S2: *continued.*

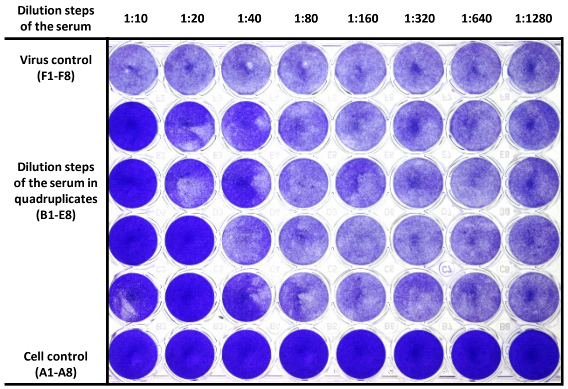

Routine 4* (27 d.a.P.):
$PRNT_{50}$: 1:20.

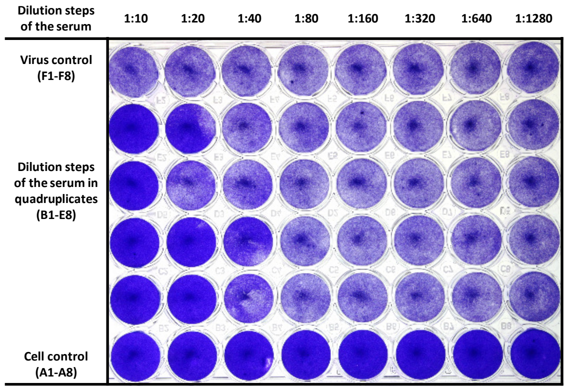

Routine 4* (153 d.a.P.):
$PRNT_{50}$: 1:20.

d.a.P., days after positive PCR test
* with respect to SARS-CoV-2 patient 14

Figure S2: *continued.*

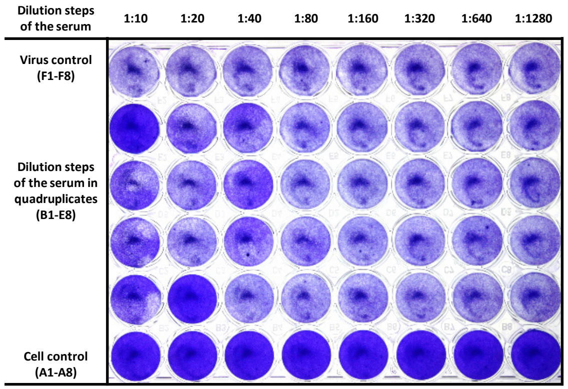

Routine 5* (27 d.a.P.):
$PRNT_{50}$: ≤ 1:10.

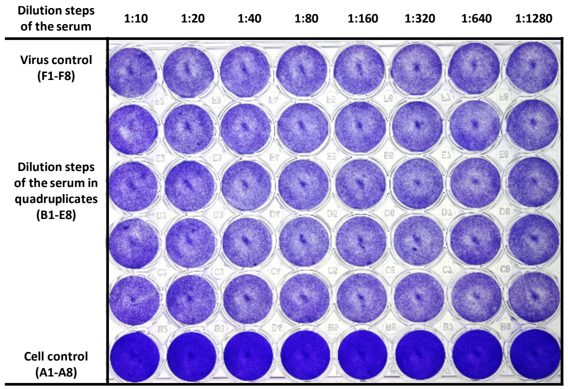

Routine 5* (156 d.a.P.):
$PRNT_{50}$: < 1:10.

d.a.P., days after positive PCR test
* with respect to SARS-CoV-2 patient 14

Figure S2: *continued.*

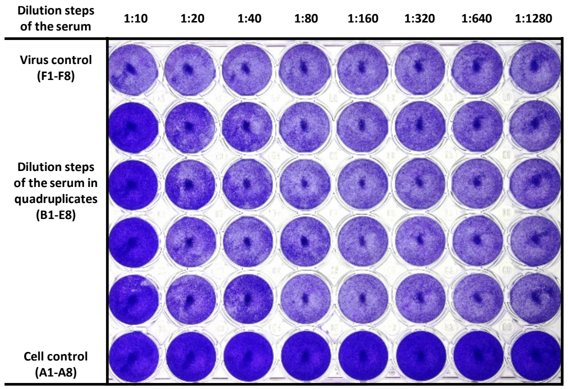

Routine 6* (27 d.a.P.):
$PRNT_{50}$: 1:10.

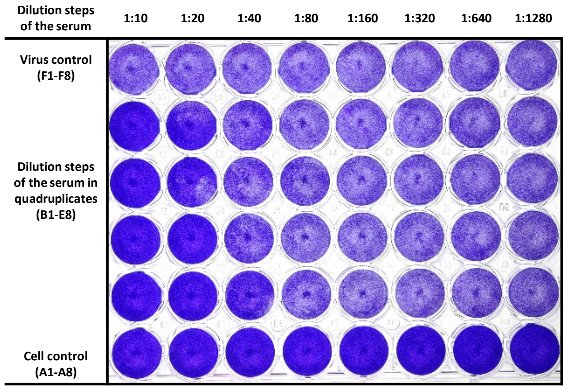

Routine 6* (92 d.a.P.):
$PRNT_{50}$: 1:20.

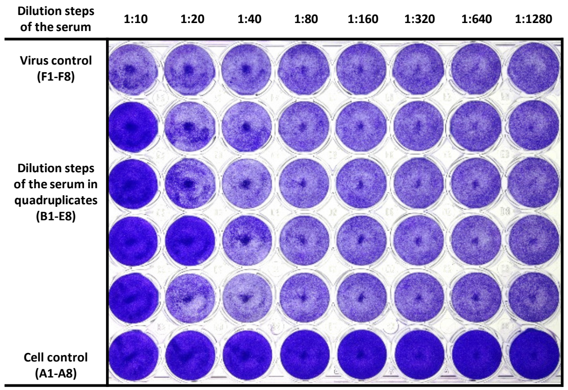

Routine 6* (127 d.a.P.):
$PRNT_{50}$: 1:10.

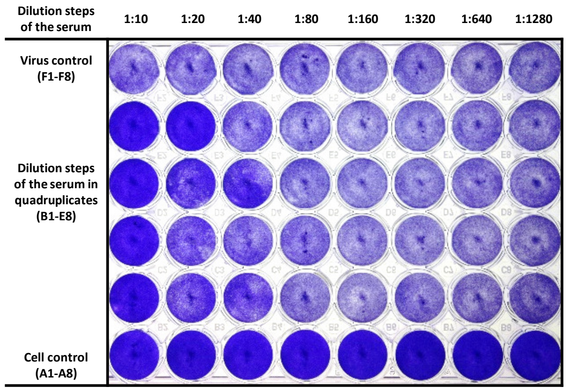

Routine 6* (153 d.a.P.):
$PRNT_{50}$: 1:10.

d.a.P., days after positive PCR test
* with respect to SARS-CoV-2 patient 14

Figure S2: *continued.*

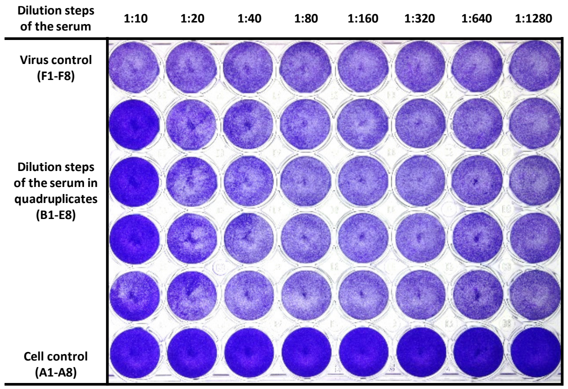

Routine 13* (92 d.a.P.):
$PRNT_{50}$: 1:10.

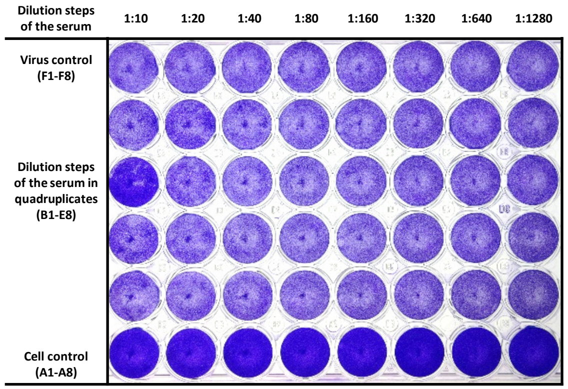

Routine 13* (127 d.a.P.):
$PRNT_{50}$: < 1:10.

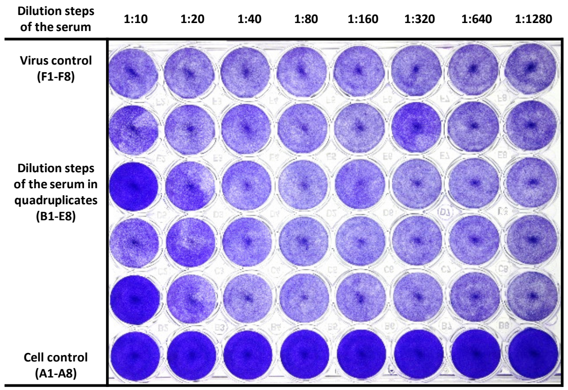

Routine 13* (153 d.a.P.):
$PRNT_{50}$: ≤ 1:10.

d.a.P., days after positive PCR test
* with respect to SARS-CoV-2 patient 14

Figure S2: *continued.*

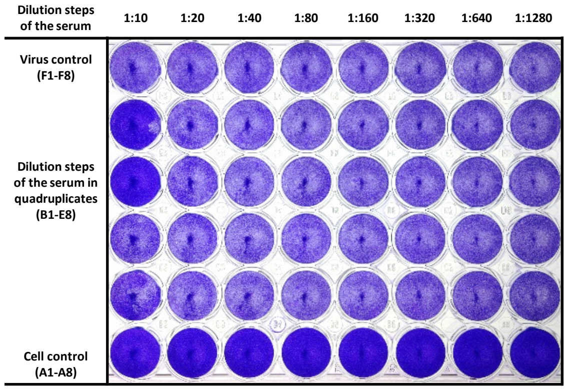

Routine 8 (0 d.a.P.):
$PRNT_{50}$: ≤ 1:10.

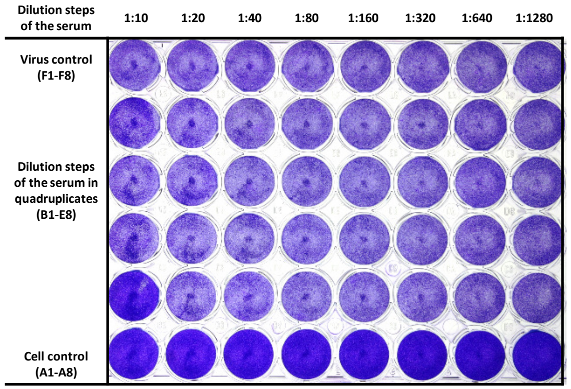

Routine 8 (112 d.a.P.):
$PRNT_{50}$: < 1:10.

d.a.P., days after positive PCR test
