# Supplementary material for: Kinetics of Nucleo- and Spike Protein-Specific Immunoglobulin G and of Virus-Neutralizing Antibodies after SARS-CoV-2 Infection
Source: Microorganisms. 2020 Oct 13;8(10):1572. doi: 10.3390/microorganisms8101572 (PMC7650537; doi:10.3390/microorganisms8101572)
Supplement: Supplementary file 1 [file microorganisms-08-01572-s001.zip › supplementary_material/Figure_S3_revised.pdf]

Figure S3: Development of high SARS-CoV-2 IgG avidities in three SARS-CoV-2 patients.

SARS-CoV-2
Patient 1

						ReaktKontr(0), IgG(1), IgA(2), IgM(3), CutOff(4), NP_229E(5), NP_NL63(6), NP_OC43(7), NP_HKU1(8), NP_SARS_2(9), RBD_SARS_2(10), S1_SARS_2(11)	
4 d.a.P.	1	Patient	IgG		42110507	0 1 4 6 7 8	NP_OC43(3,8); NP_HKU1(3,3)
6 d.a.P.	1	Patient	IgG		42111625	0 1 4 5 6 7 8 9	NP_229E(1,1); NP_NL63(1,8); NP_OC43(4,1); NP_HKU1(4,4)
	2	Avidität	IgG		42111625	0 1 4 5 6 7 8	NP 229E(0.70=h);NP_NL63(0.71=h);NP_OC43(0.60=n);NP_HKU1(0.48=n)
8 d.a.P.	2	Patient	IgG		42114145	0 1 3 4 5 6 7 8 9 10 11	NP_NL63(2,2); NP_OC43(3,2); NP_HKU1(2,8); NP_SARS_2(3,4); RBD_SARS_2(1,9); S1_SARS_2(2,4)
11 d.a.P.	3	Patient	IgG		42115601	0 1 4 5 6 7 8 9 10 11	NP 229E(2,0); NP NL63(2,5); NP OC43(2,3); NP HKU1(2,4); NP_SARS_2(6,3); RBD_SARS_2(6,8); S1_SARS_2(7,1)
	4	Avidität	IgG		42115601	0 1 4 5 6 7 8 9 10 11	NP 229E(0.59=n);NP NL63(0.85=h);NP OC43(0.68=h);NP HKU1(0.62=h);NP_SARS_2(0.11=n);RBD_SARS_2(0.31=n);S1_SARS_2(0.24=n)
15 d.a.P.	1	Patient	IgG		42120066	0 1 4 5 6 7 8 9 10 11	NP_229E(2,1); NP_NL63(3,0); NP_OC43(3,3); NP_HKU1(3,0); NP_SARS_2(6,4); RBD_SARS_2(6,8); S1_SARS_2(7,0)
	2	Avidität	IgG		42120066	0 1 4 5 6 7 8 9 10 11	NP_229E(0.28=n);NP_NL63(0.79=h);NP_OC43(0.50=n);NP_HKU1(0.45=n);NP_SARS_2(0.11=n);RBD_SARS_2(0.24=n);S1_SARS_2(0.21=n)
26 d.a.P.	1	Patient	IgG		42127890	0 1 4 5 6 7 8 9 10 11	NP 229E(3,0); NP NL63(3,7); NP OC43(4,9); NP HKU1(3,9); NP_SARS_2(9,6); RBD_SARS_2(10,1); S1_SARS_2(10,2)
	2	Avidität	IgG		42127890	0 1 4 5 6 7 8 9 10 11	NP 229E(0.30=n);NP NL63(0.69=h);NP OC43(0.42=n);NP HKU1(0.44=n);NP_SARS_2(0.14=n);RBD_SARS_2(0.28=n);S1_SARS_2(0.29=n)
102 d.a.P.	19	Patient	IgG		42222720	0 1 4 5 6 7 8 9 10 11	NP_229E(1,8); NP_NL63(2,8); NP_OC43(3,6); NP_HKU1(2,6); NP_SARS_2(10,1); RBD_SARS_2(10,1); S1_SARS_2(10,2)
	20	Avidität	IgG		42222720	0 1 4 5 6 7 8 9 10 11	NP_229E(0.43=n);NP_NL63(0.70=h);NP_OC43(0.52=n);NP_HKU1(0.56=n);NP_SARS_2(0.77=h);RBD_SARS_2(0.93=h);S1_SARS_2(0.93=h)
132 d.a.P.	1	Avidität	IgG		42264860	0 1 4 6 7 8 9 10 11	NP_229E(0.00=n);NP_NL63(0.31=n);NP_OC43(0.27=n);NP_HKU1(0.38=n);NP_SARS_2(0.40=n);RBD_SARS_2(0.76=h);S1_SARS_2(0.78=h)
	2	Patient	IgG		42264860	0 1 4 5 6 7 8 9 10 11	NP_229E(1,2); NP_NL63(2,2); NP_OC43(2,4); NP_HKU1(1,6); NP_SARS_2(10,5); RBD_SARS_2(10,3); S1_SARS_2(10,1)
161 d.a.P.	1	Patient	IgG		42308810	0 1 4 5 6 7 8 9 10 11	NP_NL63(1,6); NP_OC43(2,6); NP_HKU1(1,7); NP_SARS_2(7,2); RBD_SARS_2(6,9); S1_SARS_2(6,8)
	2	Avidität	IgG		42308810	0 1 4 5 6 7 8 9 10 11	NP_NL63(0.74=h);NP_OC43(0.47=n);NP_HKU1(0.36=n);NP_SARS_2(0.60=n);RBD_SARS_2(0.86=h);S1_SARS_2(0.90=h)

d.a.P, days after the positive SARS-CoV-2 PCR

Figure S3: *continued.*

ReaktKontr(0) | IgG(1) | IgA(2) | IgM(3) | CutOff(4) | NP_229E(5) | NP_NL63(6) | NP_OC43(7) | NP_HKU1(8) | NP_SARS_2(9) | RBD_SARS_2(10) | S1_SARS_2(11)

SARS-CoV-2
Patient 18

d.a.P.	No.	Test	Ig	ID	Strip bands	Result
26 d.a.P.	1	Avidität	IgG	42136433	0 1 4 9 10 11	NP_SARS_2(0.39=n);RBD_SARS_2(0.29=n);S1_SARS_2(0.33=n)
	2	Patient	IgG	42136433	0 1 2 4 9 10 11	NP_SARS_2(6,4); RBD_SARS_2(4,9); S1_SARS_2(6,1)
46 d.a.P.	3	Avidität	IgG	42158360	0 1 2 4 9 10 11	NP_SARS_2(0.16=n);RBD_SARS_2(0.20=n);S1_SARS_2(0.43=n)
	4	Patient	IgG	42158360	0 1 2 4 8 9 10 11	NP_SARS_2(3,0); RBD_SARS_2(4,6); S1_SARS_2(5,7)
91 d.a.P.	5	Avidität	IgG	42221386	0 1 4 5 6 9 10 11	NP 229E(0.70=h);NP NL63(0.82=h);NP_SARS_2(0.17=n);RBD_SARS _2(0.17=n);S1_SARS_2(0.24=n)
	6	Patient	IgG	42221386	0 1 4 5 6 9 10 11	NP 229E(1,3); NP_NL63(1,8); NP_SARS_2(5,1); RBD_SARS_2(2,8); S1_SARS_2(3,3)
130 d.a.P.	9	Avidität	IgG	42271849	0 1 4 9 10 11	NP_SARS_2(0.35=n);RBD_SARS_2(0.83=h);S1_SARS_2(0.79=h)
	10	Patient	IgG	42271849	0 1 4 9 10 11	NP_SARS_2(4,5); RBD_SARS_2(4,4); S1_SARS_2(5,1)
147 d.a.P.	3	Patient	IgG	42299533	0 1 2 4 7 8 9 10 11	NP_SARS_2(6,6); RBD_SARS_2(6,4); S1_SARS_2(6,7)
	4	Avidität	IgG	42299533	0 1 2 4 8 9 10 11	NP_SARS_2(0.29=n);RBD_SARS_2(0.68=h);S1_SARS_2(0.71=h)

SARS-CoV-2
Patient 21

d.a.P.	No.	Test	Ig	ID	Strip bands	Result
19 d.a.P.	5	Patient	IgG	42141894	0 1 4 5 6 7 8 9 10 11	NP_OC43(1,1); NP_HKU1(1,7); NP_SARS_2(5,7); RBD_SARS_2(3,5); S1_SARS_2(3,9)
	6	Avidität	IgG	42141894	0 1 4 5 7 8 9 10 11	NP_OC43(0.25=n);NP_HKU1(0.52=n);NP_SARS_2(0.34=n);RBD_SARS _2(0.20=n);S1_SARS_2(0.16=n)
65 d.a.P.	23	Patient	IgG	42199832	0 1 4 5 6 7 8 9 10 11	NP_229E(1,2); NP_NL63(1,7); NP_OC43(1,9); NP_HKU1(2,1); NP_SARS_2(8,0); RBD_SARS_2(6,2); S1_SARS_2(6,7)
	24	Avidität	IgG	42199832	0 1 4 5 6 7 8 9 10 11	NP_229E(0.67=h);NP_NL63(0.40=n);NP_OC43(0.25=n);NP_HKU1(0.6 9=h);NP_SARS_2(0.88=h);RBD_SARS_2(0.61=h);S1_SARS_2(0.55=n)
104 d.a.P.	1	Avidität	IgG	42254953	0 1 4 8 9 10 11	NP_HKU1(0.26=n);NP_SARS_2(0.66=h);RBD_SARS_2(0.41=n);S1_SA RS_2(0.46=n)
	2	Patient	IgG	42254953	0 1 4 5 6 7 8 9 10 11	NP_HKU1(1,6); NP_SARS_2(7,5); RBD_SARS_2(5,4); S1_SARS_2(5,4)
121 d.a.P.	3	Patient	IgG	42277754	0 1 4 5 6 7 8 9 10 11	NP_229E(1,0); NP_NL63(1,5); NP_OC43(1,9); NP_HKU1(2,0); NP_SARS_2(7,6); RBD_SARS_2(6,2); S1_SARS_2(6,3)
	4	Avidität	IgG	42277754	0 1 4 5 6 7 8 9 10 11	NP_229E(0.53=n);NP_NL63(0.32=n);NP_OC43(0.20=n);NP_HKU1(0.5 0=n);NP_SARS_2(0.88=h);RBD_SARS_2(0.52=n);S1_SARS_2(0.52=n)

d.a.P, days after the positive SARS-CoV-2 PCR
