# Supplementary material for: Kinetics of Nucleo- and Spike Protein-Specific Immunoglobulin G and of Virus-Neutralizing Antibodies after SARS-CoV-2 Infection
Source: Microorganisms. 2020 Oct 13;8(10):1572. doi: 10.3390/microorganisms8101572 (PMC7650537; doi:10.3390/microorganisms8101572)
Supplement: Supplementary file 1 [file microorganisms-08-01572-s001.zip › supplementary_material/Table_S1.pdf]

Table S1: Characteristics of the eight SARS-CoV-2 IgG/total antibody tests.

**Assay**	**Manufacturer**	**Assay principle**	**Immunoglobuline (Ig) class**	**SARS-CoV-2 antigen(s)**
SARS-CoV-2-IgG-Assay	Abbott	chemiluminescent microparticle immunoassay	IgG	nucleoprotein
LIAISON® SARS-CoV-2 S1/S2 IgG	Diasorin	chemiluminescence immunoassay	IgG	spike protein units S1&S2
EDI™ Novel Coronavirus COVID-19 IgG ELISA Kit	Epitope Diagnostics	enzyme-linked immuno sorbent assay	IgG	nucleoprotein
SARS-CoV-2-ELISA	Euroimmun	enzyme-linked immuno sorbent assay	IgG	spike protein unit S1
*recom*Well SARS-CoV-2 IgG	Mikrogen	enzyme-linked immuno sorbent assay	IgG	nucleoprotein
Elecsys Anti-SARS-CoV-2	Roche	electro-chemiluminescence immunoassay	total antibodies	nucleoprotein
SARS-CoV-2 ViraChip® IgG	Viramed	microarray	IgG	spike protein units S1&S2, nucleoprotein
SERION ELISA agile SARS-COV-2 IgG	Virion-Serion	enzyme-linked immuno sorbent assay	IgG	whole spike protein
