# Supplementary figures and images for: Kinetics of Nucleo- and Spike Protein-Specific Immunoglobulin G and of Virus-Neutralizing Antibodies after SARS-CoV-2 Infection
Source: Microorganisms. 2020 Oct 13;8(10):1572. doi: 10.3390/microorganisms8101572 (PMC7650537; doi:10.3390/microorganisms8101572)

**Figure S4:** Agreement of titers from two independent neutralization tests.

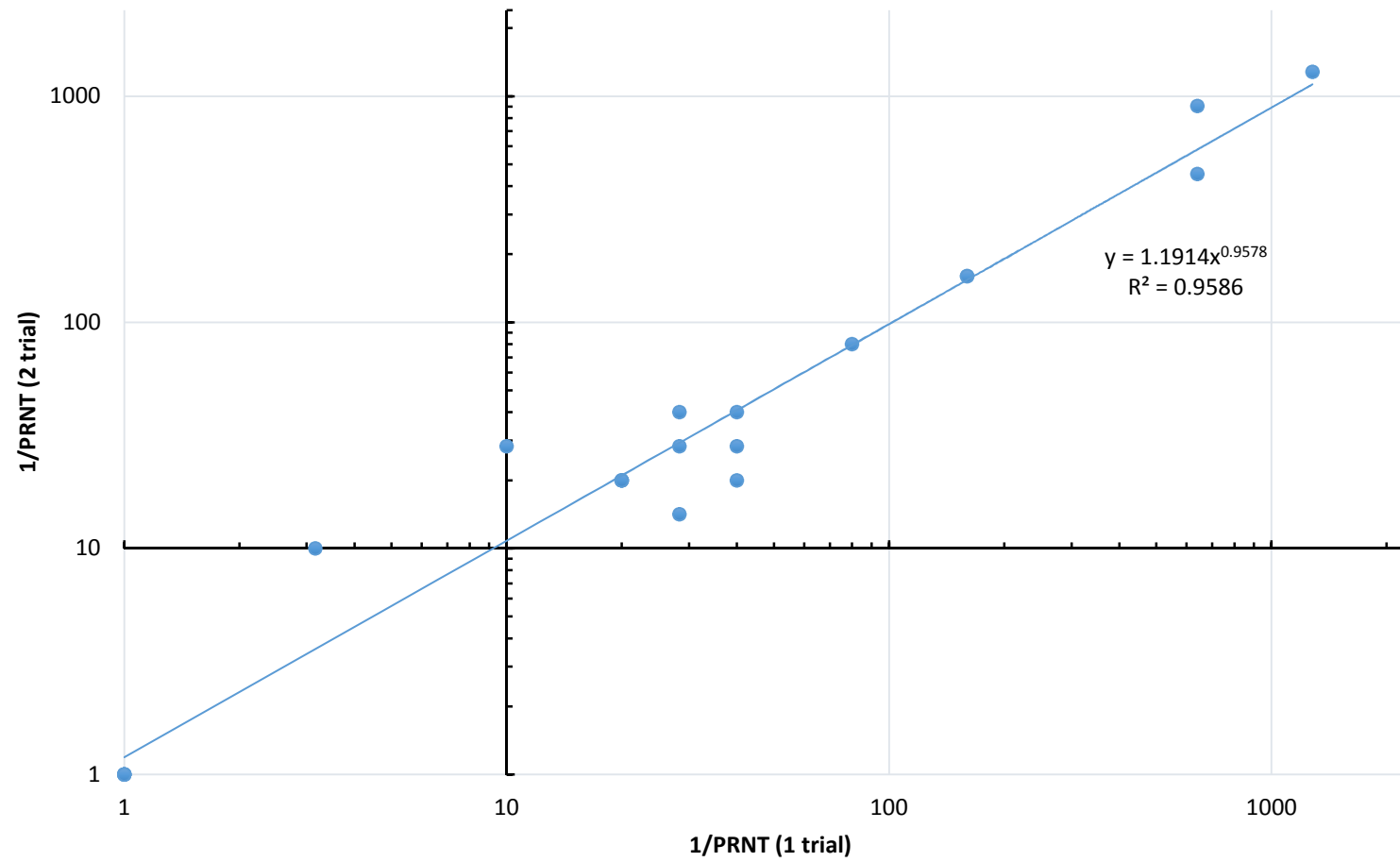

Supplement: Supplementary file 1 [file microorganisms-08-01572-s001.zip › supplementary_material/Figure_S4_revised.pdf]
